# Metal exchange in lithiocuprates: implications for our understanding of structure and reactivity†
†Electronic supplementary information (ESI) available. CCDC 1540280–1540286. For ESI and crystallographic data in CIF or other electronic format see DOI: 10.1039/c7sc01423f



**DOI:** 10.1039/c7sc01423f

**Published:** 2017-05-04

**Authors:** Andrew J. Peel, Ryan Ackroyd, Andrew E. H. Wheatley

**Affiliations:** a Department of Chemistry , University of Cambridge , Lensfield Road , Cambridge , CB2 1EW UK . Email: aehw2@cam.ac.uk ; Fax: +44(0) 1223 336362

## Abstract

New reagents have been sought for directed *ortho* cupration in which the use of cyanide reagents is eliminated. CuOCN reacts with excess TMPLi (TMP = 2,2,6,6-tetramethylpiperidide) in the presence of limited donor solvent to give crystals that are best represented as (TMP)_2_Cu_0.1_Li_0.9_(OCN)Li_2_(THF) **8**, whereby both Lipshutz-type lithiocuprate (TMP)_2_Cu(OCN)Li_2_(THF) **8a** and trinuclear (TMP)_2_(OCN)Li_3_(THF) **8b** are expressed. Treatment of a hydrocarbon solution of TMP_2_CuLi **9a** with LiOCN and THF gives pure **8a**. Meanwhile, formation of **8b** is systematized by reacting (TMPH_2_)OCN **10** with TMPH and *n*BuLi to give (TMP)_2_(OCN)Li_3_(THF)_2_ **11**. Important to the attribution of lower/higher order bonding in lithiocuprate chemistry is the observation that in crystalline **8**, amide-bridging Cu and Li demonstrate clear preferences for di- and tricoordination, respectively. A large excess of Lewis base gives an 8-membered metallacycle that retains metal disorder and analyses as (TMP)_2_Cu_1.35_Li_0.65_ **9** in the solid state. NMR spectroscopy identifies **9** as a mixture of (TMP)_2_CuLi **9a** and other copper-rich species. Crystals from which the structure of **8** was obtained dissolve to yield evidence for **8b** coexisting in solution with *in situ*-generated **9a**, **11** and a kinetic variant on **9a** (***i*-9a**), that is best viewed as an agglomerate of TMPLi and TMPCu. Moving to the use of DALi (DA = diisopropylamide), (DA)_2_Cu_0.09_Li_0.91_(Br)Li_2_(TMEDA)_2_ **12** (TMEDA = *N*,*N*,*N*′,*N*′-tetremethylethylenediamine) is isolated, wherein (DA)_2_Cu(Br)Li_2_(TMEDA)_2_ **12a** exhibits lower-order Cu coordination. The preparation of (DA)_2_Li(Br)Li_2_(TMEDA)_2_ **12b** was systematized using (DAH_2_)Br, DAH and *n*BuLi. Lastly, metal disorder is avoided in the 2 : 1 lithium amide : Lipshutz-type monomer adduct (DA)_4_Cu(OCN)Li_4_(TMEDA)_2_ **13**.

## Abstract 2

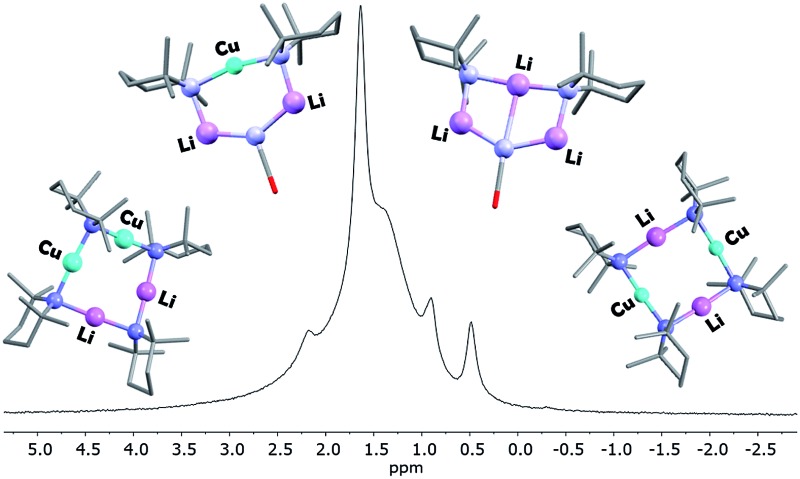
A new class of lithium cyanatocuprates are elucidated whose structures reveal a preference by copper for lower order structure formation.

## Introduction

Interest in methods for refining the regioselective functionalization of aromatics by using more selective bases has grown significantly since the inception^1^ of what have become known as ‘synergic bases’.^2^ These heterobimetallic systems – in their simplest form R_*m*_M(NR′_2_)_*n*_AM (R = organyl; *m* = 0, 2, 3; M = less polarizing metal; NR′_2_ = amide; *n* = 1, 2, 3; AM = more polarizing (alkali) metal) – have afforded new levels of reactivity,^3^ regioselectivity^4^ and functional group tolerance^5^ not hitherto available through traditional main group organometallic bases. Recently, a fruitful area of synergic base chemistry that has developed has involved directed cupration using lithium cuprate reagents.^6^ The structural variability manifest in lithium cuprate chemistry, which derives in part from the potential to enhance reactivity by introducing an alkali metal salt, highlights the need to better understand structural variability in synergic systems. The corresponding elucidation of synergic bases was initially dominated by crystallographic determination.^7^ However, their heterobimetallic composition has led to the need for solution analysis to establish the nature of competition between structure retention of the heterobimetallic moiety *versus* the cooperative action of individually monometallic reagents^8^ and the permutations for dynamic deaggregation/recombination.^7c,9^

**Scheme 1:**
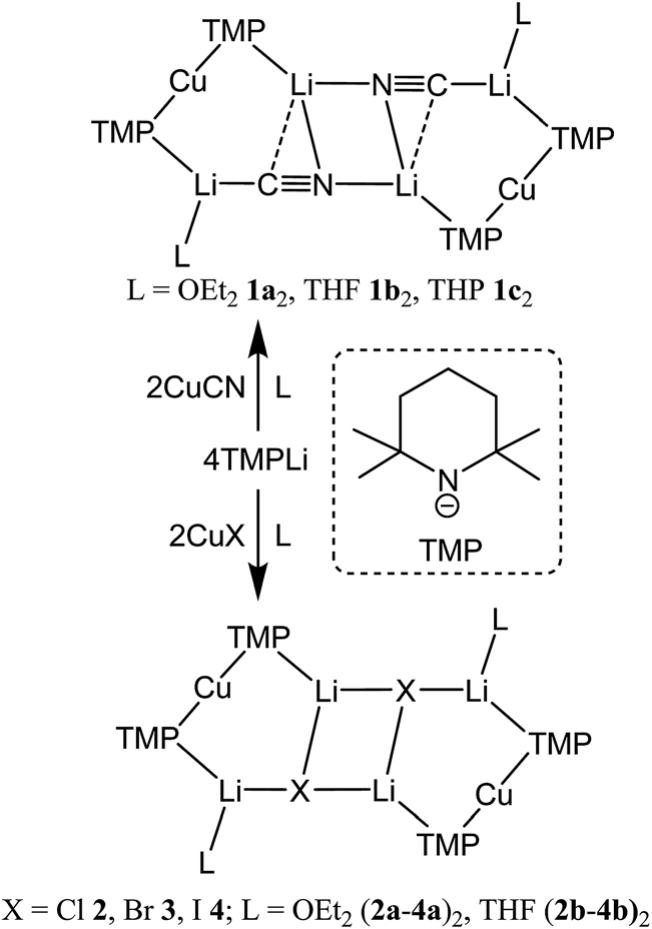
Formation of the dimers of cyano- and halogenocuprates **1–4**.

**Scheme 2:**
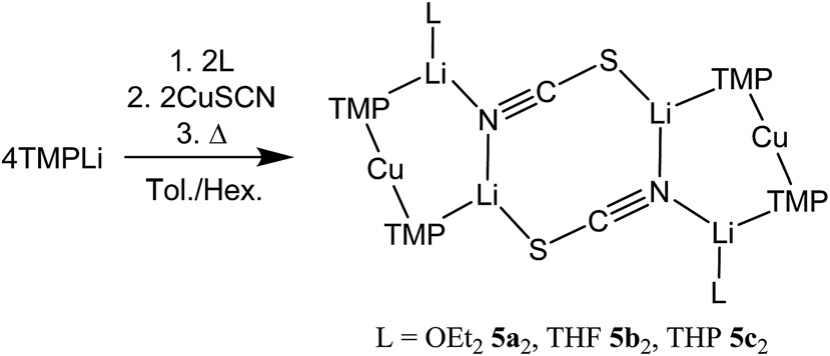
Formation of the dimers of thiocyanatocuprates **5**.

First developed 50 years ago,^10^ lithium cuprates have been subsequently modified in two main ways. First, lithium amidocuprates have been developed, offering often unique reactivities as well as the potential of the amido group as a non-transferable ligand and as a chiral auxiliary.^9,11^ Second, yield enhancements have been sought by combining so-called Gilman lithium cuprates (R_2_CuLi) with lithium salts. For the deployment of LiCN as the latter,^12^ the resulting cuprates (R_2_Cu(CN)Li_2_) were coined Lipshutz cuprates and spectroscopic studies suggested the possibility that transfer of cyanide to Cu would give a higher order (tricoordinate) metal.^13^ However, the alternative of retention of lower order (dicoordinate) copper was suggested on theoretical,^14^ spectroscopic^15^ and, more recently, X-ray diffraction^16^ grounds. The isolation and characterization of lithium cyanocuprates **1a–c**, which were prepared using amidolithium reagents with CuCN, revealed the bonding characteristics of Cu in systems that could demonstrate either higher or lower order structural properties. In line with theoretical expectations,^14^ lower order (TMP)_2_Cu(CN)Li_2_(L) (L = OEt_2_, THF, THP = tetrahydropyran)^17,18^ structures were observed crystallographically, with agglomeration giving essentially planar dimers based on lithium–nitrogen cores (Scheme 1). The observation that, whilst in other respects **1a–c** pertained to Lipshutz cuprate characteristics, they lack a Cu–CN interaction led to investigation of the generality of this motif. To this end (TMP)_2_Cu(X)Li_2_(L) (X = Cl **2**, Br **3**, I **4**, L = OEt_2_ **2a–4a**, THF **2b–4b**) were isolated (Scheme 1).^19,20^ As with the previously reported cyanide structures, these revealed lower order Cu in the solid state. The extension of monoatomic bridges to the field of triatomic inorganic anions was achieved by the development of thiocyanatocuprates. These utilize SCN^–^ in place of CN^–^ and formed (TMP)_2_Cu(SCN)Li_2_(L) (L = OEt_2_ **5a**, THF **5b**, THP **5c**), which demonstrated 8-membered metallacyclic (LiSCN)_2_ cores supported by 6-membered N_3_CuLi_2_ rings in the solid state (Scheme 2). In these structures, significant solvent effects were noted through the adoption of either planar, boat or chair conformers for the 8-membered core depending on whether the cuprate was additionally stabilized by OEt_2_, THF or THP, respectively.^21^

The recent observation that cyanide-incorporating Lipshutz cuprates exhibit solvent-controlled conversion to their Gilman counterparts^7c,h^ by the abstraction of LiCN^22^ has been superceded by the discovery that this process is likely to be responsible for the *in situ* creation of active species in solution.^23^ This has reinvigorated interest in the properties of lithium amidocuprates and it has been noted that the ability of bimetallics of the type (R_2_N)_2_CuLi to dimerize, coupled with the acknowledged issue of metal exchange between Li and Cu, as elucidated by van Koten and co-workers,^16a,b,24^ means that the field of Gilman cuprate chemistry is closely interwoven with that of copper–nitrogen metallacycles. These have been observed to constitute tetrameric ketimides,^25^ hydrazides,^25^ phosphinimides^26^ mixed amides/guanidinates^27^ and amides^9^ with the last of these ligand-types also supporting 3D clusters.^28^ Copper amides, first prepared a century ago^29^ and more recently the focus of intense study,^11a,30^ have proven to be synthetically important, with solid state tetramers shown to deaggregate and participate in modified Ullmann amination^31^ with arenes in the presence of 1,10-phenanthroline.^9^ Meanwhile, Gilman lithium amidocuprates have been established to constitute the active species in directed aromatic cupration.^17^ However, synthetic studies having established that as-prepared Gilman cuprates are ineffectual, the need to convert Lipshutz(-type) cuprates to their Gilman counterparts in solution has been shown.^23^

The current work sees further efforts to study the use of polyatomic inorganic salts in lithium amidocuprate chemistry, through the deployment of the rarely used cyanate ligand. This accesses a range of new lithium cyanatocuprates. Metal disorder in some examples of these new materials sheds important new light on the predilection of Cu for lower/higher order structure formation. Advances are also reported in our understanding of the solution behaviour of lithium cuprates.

## Results and discussion

### Synthesis and solid state analysis

**Scheme 3:**
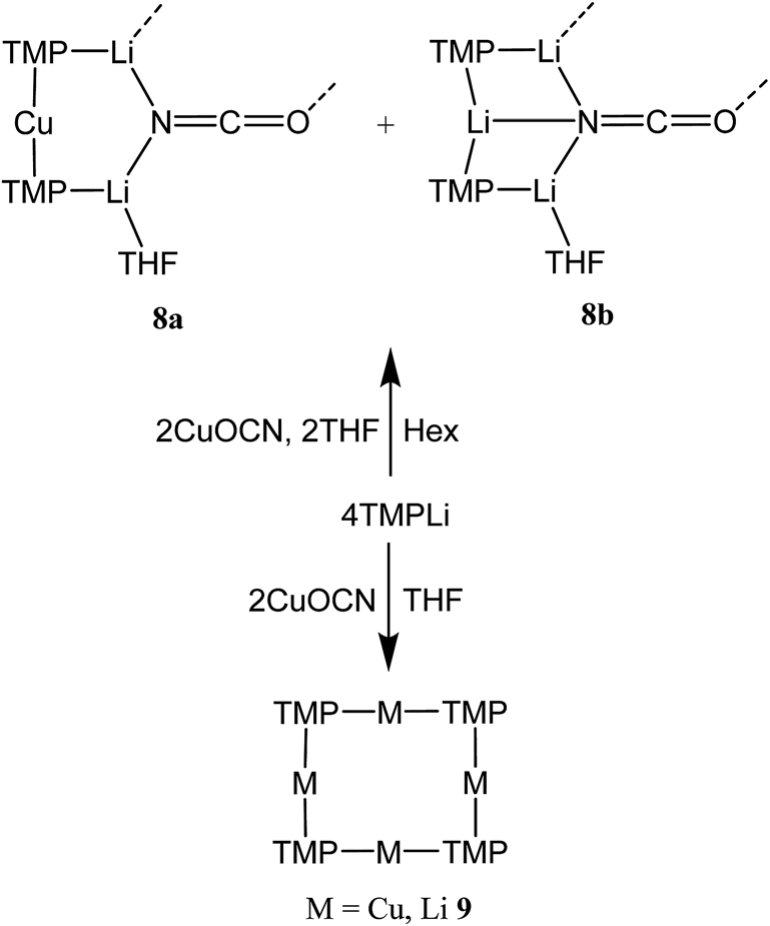
Solvent-dependent synthesis of **8a**/**b** and **9**.

**Fig. 1:**
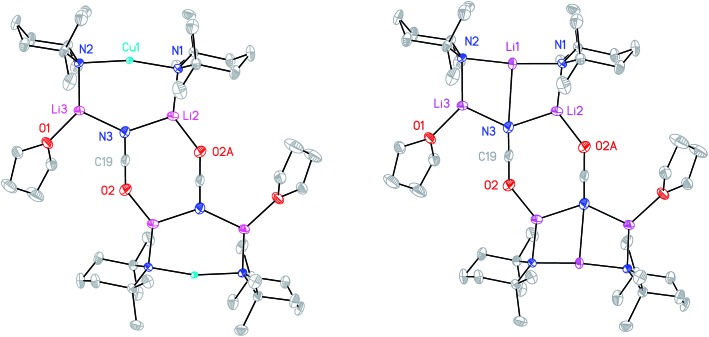
Molecular structures of **8a**_2_ (left) and **8b**_2_ (right, 30% probability and with H-atoms omitted). Selected bond lengths (Å) and angles (°): N1–Cu1 1.968(11), N2–Cu1 2.029(11), N3···Cu1 2.93(1), N1–Li1 1.970(16), N2–Li1 2.009(16), N3–Li1 2.597(11), N1–Li2 1.969(3), N2–Li3 1.935(3), N3–Li2 2.056(4), N3–Li3 2.040(4), O2–Li2A 1.904(3), O2–C19 1.211(3), N3–C19 1.168(3), N1–Cu1–N2 167.3(4), N1–Li1–N2 173.4(6), N1–Li1–N3 94.5(5), N2–Li1–N3 92.1(5).

Cu(NO_3_)_2_(H_2_O)_3_ was reacted sequentially with LiOAc(H_2_O)_2_, and LiOCN (see ESI†). The addition of SO_2_ gave a green solution from which CuOCN **7** precipitated.^32^ The ability to prepare **7** offers the possibility of analyzing the cyanate analogue of recently reported (TMP)_2_Cu(SCN)Li_2_(THF).^21^ A hexane solution of TMPLi was therefore reacted with CuOCN in a 2 : 1 ratio in the presence of THF (1 eq. wrt Cu). This gave a modest yield of crystalline blocks. IR spectroscopy showed a strong peak at 2208 cm^–1^ and X-ray diffraction pointed to the formulation (TMP)_2_M(OCN)Li_2_(THF) but revealed disorder at the TMP-bridging metal site. The optimal crystallographic refinement suggests M = Cu_0.1_Li_0.9_, and therefore a 1 : 9 co-crystalline mixture of (TMP)_2_Cu(OCN)Li_2_(THF) **8a** and (TMP)_2_(OCN)Li_3_(THF) **8b** (Scheme 3 and Fig. 1).

The X-ray diffraction structure of **8** reveals dimerization based on an 8-membered metallacyclic (LiOCN)_2_ core that incorporates a 3-coordinate Li centre (Li2). This core is supported by two 6-membered N_3_MLi_2_ rings, within which the coordination sphere of Li3 is completed by THF. However, in contrast to the previously noted THF-solvated thiocyanate **5b**_2_,^21^ **8**_2_ reveals not a boat conformation but an essentially planar arrangement, with only TMP and THF deviating significantly from a plane defined by the three conjoined metallacycles and the O(THF)–Li bonds (ESI, Fig. S1†). Of most interest is the observation that whereas the cores of **8a**_2_ and **8b**_2_ are essentially identical, the geometries of the 6-membered N_3_MLi_2_ rings vary significantly. In **8a** (M = Cu) the obtuse angle of 167.3(4)° at Cu1 results in a non-bonding N3···Cu1 distance of 2.93(1) Å. Insofar as this distance is consistent with a dicoordinate (lower) order Cu centre this bonding pattern is consistent with the recent characterization of Lipshutz cuprates **1a–c**,^17,18,21^ halogenocuprates **2–4** (ref. 19 and 20) and thiocyanatocuprates **5a–c**.^21^ In contrast, Li1 in the N_3_Li_3_ metallacycle of **8b** is located significantly closer to N3 than is Cu1 in **8a**, resulting in a reflex N1–Li1–N2 angle of 186.6(5)°. The observation of a N3–Li1 2.597(11) Å distance suggests a weak interaction between these two centres and yields a motif reminiscent of intercepted ladder-type structures reported in the past for amidolithium aggregates.^33^

**Scheme 4:**
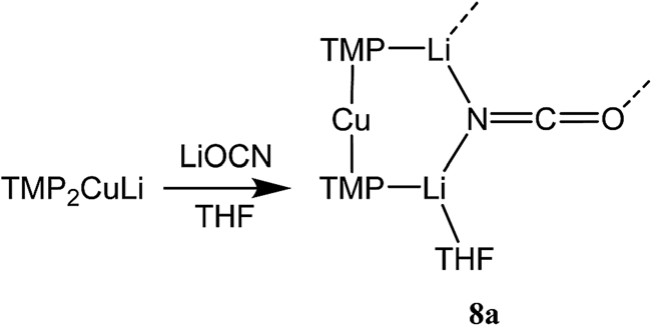
Synthesis of **8a** by a route that eliminates the possibility of LiOCN abstraction by TMPLi.

Reasoning that the depletion of copper in **8** may have been caused by the rapid sequestration of *in situ* formed LiOCN by TMPLi, we attempted to prepare **8a** by a route in which these two components were not present simultaneously. A solution of donor-free TMP_2_CuLi (**9a**) was therefore prepared using hydrocarbon solvent. This was combined with LiOCN and THF was introduced, later being replaced with hexane. The resulting solution gave radiating fans of crystals (Scheme 4). X-ray crystallography reveals the expected centrosymmetric dimer of **8a** (ESI, Fig. S3†). Though pure **8a**_2_ and its counterpart component of **8**_2_ are not strictly isostructural, the differences in molecular structure are small. In particular, marginally reduced planarity in pure **8a**_2_ when compared to its partner component of **8**_2_, results in a larger value of mean deviation from the plane of 0.205 Å (excluding THF and TMP-carbons, *cf.* 0.085 Å for **8a**_2_ in **8**_2_). These observations suggest that although weak, the N3···Li1 transannular interaction in component **8b**_2_ of **8**_2_ exerts an influence, *via* crystal packing, over minor component **8a**_2_ in the same structure.

**Scheme 5:**
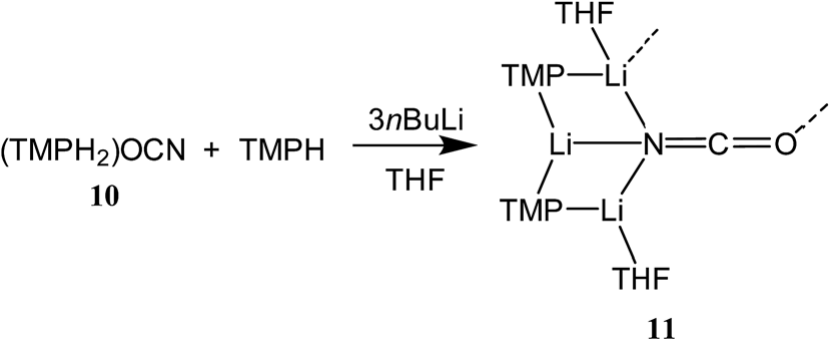
Lithiation of **10** and TMPH in bulk THF to give **11**.

**Fig. 2:**
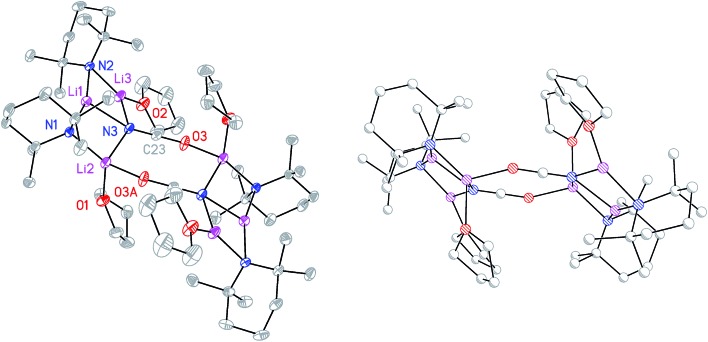
Molecular structure of **11**_2_ (30% probability and with H-atoms omitted). Selected bond lengths (Å) and angles (°): N1–Li1 2.029(3), N2–Li1 2.088(3), N3–Li1 2.198(3), N1–Li2 2.030(3), N2–Li3 1.930(3), N3–Li2 2.228(3), N3–Li3 2.036(3), O3A–Li2 1.968(3), O3–C27 1.208(2), N3–C27 1.172(2), N1–Li1–N2 163.39(16), N1–Li1–N3 100.56(12), N2–Li1–N3 95.39(12).

To attempt the synthesis of pure **8b**, two different routes were pursued. The documented use of a secondary ammonium salt to generate a mixed lithium halide–lithium amide aggregate *in situ* ^34^ led us to synthesize cyanate salt (TMPH_2_)OCN **10** (see ESI†). This was then reacted with TMPH and *n*BuLi in a 1 : 1 : 3 ratio. The deployment of bulk THF as reaction medium followed by its removal and replacement with hexane gave crystals. Alternatively, direct combination of LiOCN with TMPLi (1 : 2) in THF followed by recrystallization from hexane provided the same product. ^1^H NMR spectroscopy revealed these to comprise TMP and THF in a 1 : 1 ratio and IR spectroscopy gave a strong peak at 2207 cm^–1^. X-ray diffraction confirmed the formulation (TMP)_2_(OCN)Li_3_(THF)_2_ **11** (Scheme 5), with dimerization occurring in the same way as with **8b** (Fig. 1). The observation that this O–Li interaction is longer in **11** (O3A–Li2 1.968(3)°; Fig. 2) than in **8b** is explained by additional THF rendering Li2 tetracoordinate. The result of this THF-inclusion on the wider structure is significant. With Li2 now pseudo-tetrahedral, **11**_2_ adopts a chair conformation in which only the core (LiOCN)_2_ ring occupies a single plane. In contrast to the structure of **8**_2_ (ESI, Fig. S1†) the lithium–nitrogen heterocycles and the THF molecules now deviate significantly above or below this central plane. Peripheral to the structure core, the geometry of the (NLi)_3_ arrangement in **11** imitates that in **8b**, though the expanded N1–Li1–N2 reflex angle of 196.61(16)° allows a transannular interaction (N3–Li1 = 2.198(3) Å) shorter than that in **8b** and similar to other N–Li bonds in the structure. A further point of contrast between **8b** and **11** lies in the orientation of the amido groups. The ability of these to adopt different orientations for steric reasons has been noted previously in lithiocuprate systems.^18^ In the current work, the TMP ligands in **8b** mimic the amido groups in lithiocuprate **5b** whereby the 6-membered rings of the two TMP groups bonded to a Cu centre are each oriented away from one another and so lie flat or *endo*, *endo* with respect to the (LiOCN)_2_ structure core (Fig. 1). However, the inclusion of additional THF appears instrumental in causing one TMP ligand to adopt an upright orientation in **11**,^35^ giving an *exo*, *endo* ligand pattern instead (Fig. 2).

**Fig. 3:**
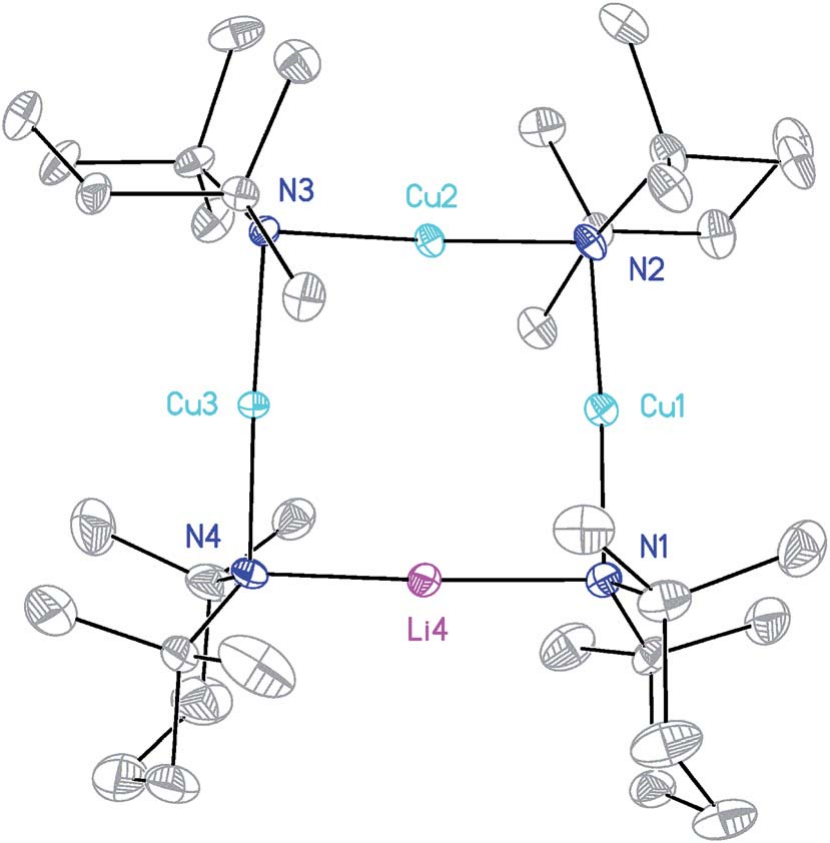
Molecular structure of **9** (30% probability and with H-atoms omitted, major metal occupancies shown). Selected bond lengths (Å): N1–M1 1.953(3), N2–M1 1.973(3), N2–M2 1.931(3), N3–M2 1.922(3), N3–M3 1.961(3), N4–M3 1.955(3), N4–M4 2.001(4), N1–M4 2.018(4).

The ability to isolate **8** proved solvent dependent; an equivalent synthesis in bulk THF (Scheme 3) yielded a crystalline material, **9**, which analyses in the solid state as an 8-membered metallacyclic copper-rich material TMP_*m*+*n*_Cu_*m*_Li_*n*_ (in **9**, *m* + *n* = 4; Fig. 3). The contribution of different species to the overall composition of **9** could only be elucidated by solution studies (see below). The structure shown in Fig. 3 can be rationalized in conjunction with the observation of that of **8**; the excess THF solvating NCOLi and abstracting it from Lipshutz-type **8** to give **9**. This view explains the deficiency of Cu in **8**.

**Scheme 6:**
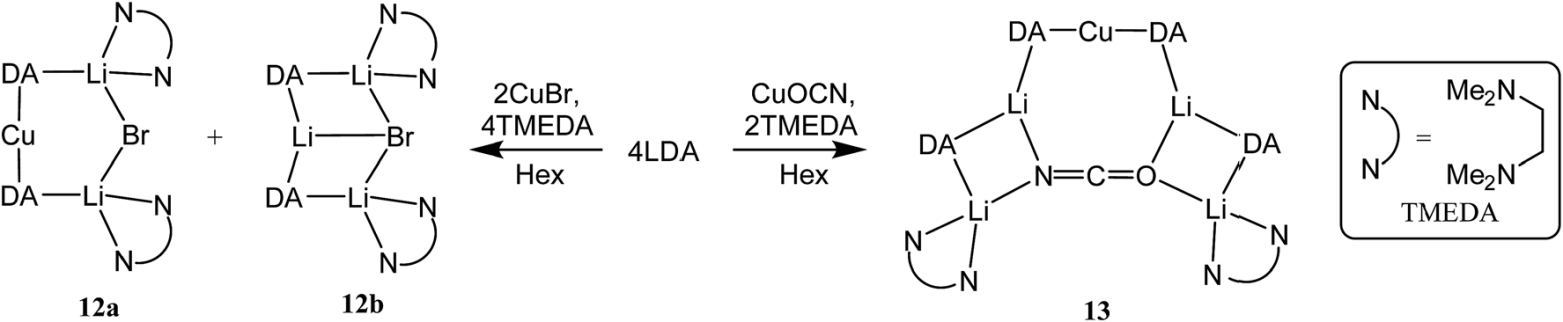
Anion-dependent synthesis of **12a**/**b** and **13**.

We next investigated the use of stronger Lewis bases as solvents for lithiocuprates. TMEDA (*N*,*N*,*N*′,*N*′-tetremethylethylenediamine) was introduced to 2 : 1 mixtures of amidolithium and Cu(i) reagents. Using DAH (= diisopropylamine), crystals were obtained incorporating different inorganic anions. As with prior work in the field of lithiocuprate chemistry, the use of CuBr as copper source enabled the isolation of a product containing both amide (in this case DA) and Lewis base (in this case TMEDA). This was achieved by treating DAH and TMEDA with *n*BuLi and CuBr in a 2 : 2 : 2 : 1 ratio in hydrocarbon media. ^1^H NMR spectroscopy suggested that the resulting isolable material incorporated DA and TMEDA in a 1 : 1 ratio, pointing to possible isolation of the Lipshutz-type cuprate (DA)_2_Cu(Br)Li_2_(TMEDA)_2_. X-ray diffraction revealed a more complex picture, displaying disorder at the amide-bridging metal site, with the best crystallographic refinement suggesting a Cu_0.09_Li_0.91_ population, *i.e.* a *ca.* 1 : 9 co-crystalline mixture of Lipshutz-type (DA)_2_Cu(Br)Li_2_(TMEDA)_2_ **12a** and (DA)_2_Li(Br)Li_2_(TMEDA)_2_ **12b** (collectively **12**, Scheme 6).

**Fig. 4:**
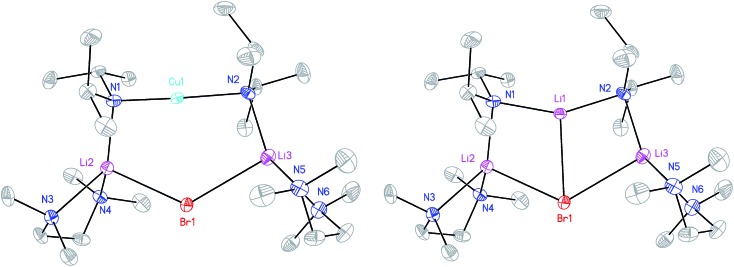
Molecular structure of the monomers of **12a** and **12b** (30% probability and with H-atoms omitted). Selected bond lengths (Å) and angles (°): N1–Cu1 1.823(13), N2–Cu1 2.064(13), Br1···Cu1 3.073(10), N1–Li1 2.009(16), N2–Li1 1.997(16), Br1–Li1 2.624(14), N1–Li2 2.047(9), N2–Li3 2.022(10), Br1–Li2 2.640(8), Br1–Li3 2.688(9), N1–Cu1–N2 176.0(7), N1–Li2–Br1 101.1(4), N2–Li3–Br1 102.5(4), Li2–Br1–Li3 125.5(3).

X-ray diffraction for **12** reveals a monomer, with the bromide bridging two TMEDA-solvated Li^+^ centres to yield a 6-membered N_2_MLi_2_Br core. For M = Cu (Fig. 4, left) this is a motif known in bis(amido)lithiocuprate chemistry, though previously the use of monodentate Lewis bases yielded dimers,^19–21^ with the exception of (Ph_2_N)_2_Cu(NPh_2_)Li_2_(OEt_2_)_2_.^11*a*^ In contrast to **8**, but in common with previous reports of Lipshutz-type bis(amido)lithiocuprates incorporating 6-membered metallacycles, the Cu centre in **12a** is essentially linear (N1–Cu1–N2 176.0(7)°) and the Cu1···Br1 distance (3.073(10) Å) is non-bonding. The dicoordinate (lower order) nature of Cu is highlighted by comparison with **12b**, in which Li1 is located significantly closer to Br1 (2.624(14) Å) than Cu1 is in **12a**. As with **8b**, this displacement of the alkali metal results in a transannular interaction, the reflex N1–Li1–N2 angle being 208.4(8)°.

**Scheme 7:**
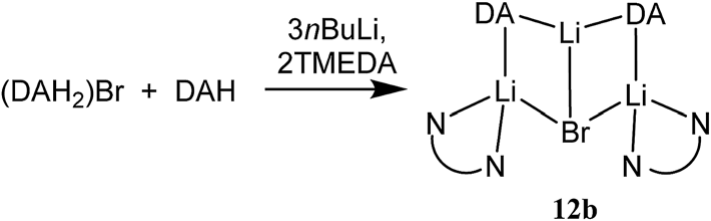
Lithiation of (DAH_2_)Br and DAH to give pure **12b**.

**Fig. 5:**
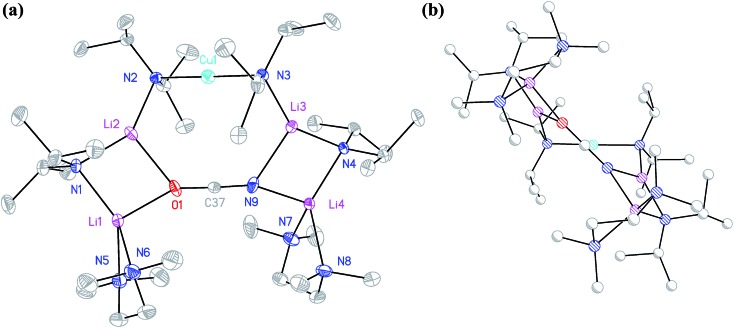
(a) Molecular structure of **13** (30% probability and with minor disorder and H-atoms omitted). Selected bond lengths (Å) and angles (°): N2–Cu1 1.910(2), N3–Cu1 1.909(2), N2–Li2 2.058(5), N1–Li2 1.991(5), N1–Li1 2.044(5), N3–Li3 2.053(5), N4–Li3 1.985(5), N4–Li4 2.048(5), N9–Li3 2.159(6), N9–Li4 2.026(5), O1–Li2 2.137(6), O1–Li1 2.009(5), N9–C37 1.202(4), O1–C37 1.205(4), N2–Cu1–N3 179.09(11), N2–Li2–O1 117.7(2), N3–Li3–N9 117.4(2), Li2–O1–C37 126.3(2), Li2–O1–Li1 81.6(2), Li2–N1–Li1 84.4(2), N1–Li1–O1 98.0(2), Li3–N9–C37 121.9(2), Li3–N9–Li4 81.3(2), Li3–N4–Li4 85.1(2), N4–Li4–N9 97.9(2); (b) **13** viewed along the C37···Cu1 vector and with the N3–Cu1–N2 unit oriented horizontally.

The structure of **12b** bears comparison with the highly unusual lithium amide–lithium chloride adduct (DA)_2_Li(Cl)Li_2_(TMEDA)_2_ (ref. 34) and, in the same way that the synthesis of amidolithium–cyanatolithium **8b** could be systematized using the cyanate salt **10**, it proved possible to achieve a synthesis of pure **12b** by reacting (DAH_2_)Br with *n*BuLi in the presence of TMEDA (Scheme 7 and ESI, Fig. S4†). The removal of metal disorder to target pure cuprate **12a** was also attempted; DAH was treated with *n*BuLi and TMEDA and then with CuOCN in hexane. The resulting crystalline material yielded NMR spectroscopic data that pointed to the presence of DA and TMEDA in a 2 : 1 ratio, while IR spectroscopy revealed a cyanate peak at 2208 cm^–1^. These data were inconsistent with the structure type exhibited by **12a** and, in due course, X-ray diffraction revealed **13** to be a 2 : 1 adduct between DALi and the cuprate (DA)_2_Cu(OCN)Li_2_(TMEDA)_2_. Crystallography pointed to a complete lack of metal disorder at the DA-bridging position (Fig. 5a). It is immediately apparent that the behaviour of the cyanate ligand contrasts with that noted in **8a**, where the bonding mode adopted by the ligand yielded both a 6-membered cuprate ring and facilitated dimerization. This variability in cyanate coordination is similar to that noted recently for the thiocyanate ligand^21^ and, in the current case, the cyanate ligand participates in the formation of an 8-membered N_2_CuLi_2_NCO ring. The capture of two molecules of DALi gives 4-membered N_2_Li_2_ and NOLi_2_ rings. Whereas **8a** incorporates an essentially flat N_2_CuLi_2_N ring, the 8-membered metallacycle in **13** deviates significantly from planarity, allowing the DALi molecules to project above and below the plane incorporating the N–Cu–N unit and the cyanate C-centre (Fig. 5b).

### NMR spectroscopy

**Fig. 6:**
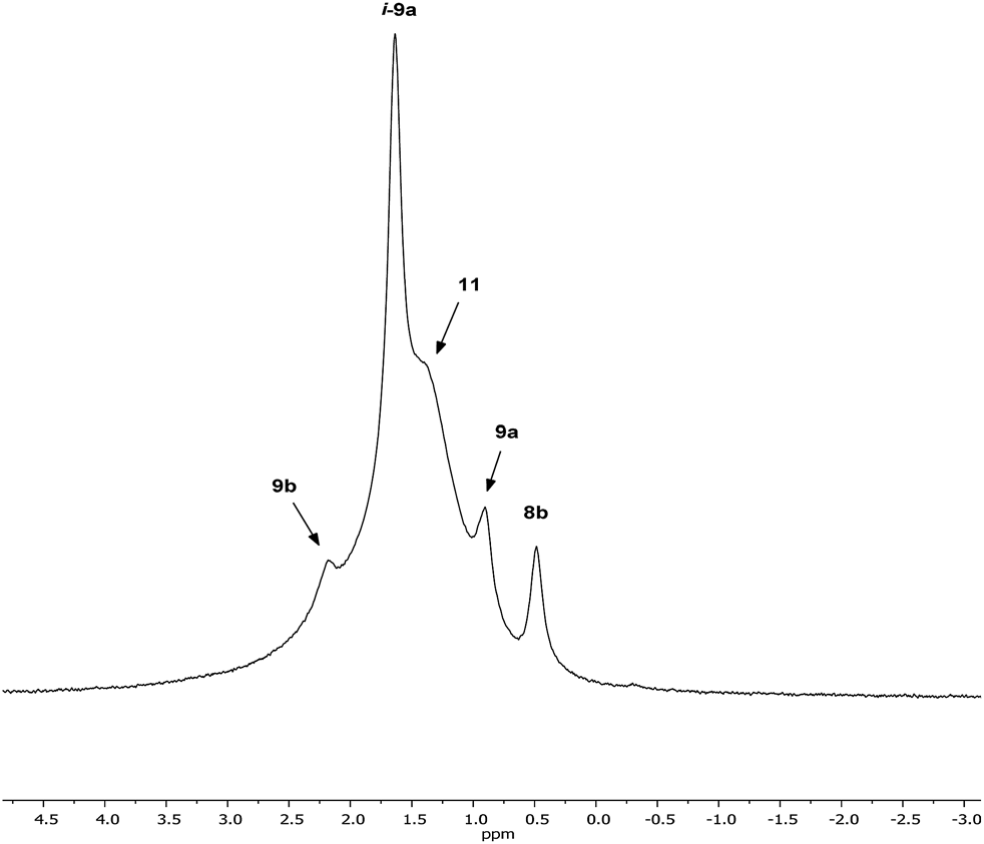
^7^Li NMR spectrum of bulk **8** in C_6_D_6_.

**Fig. 7:**
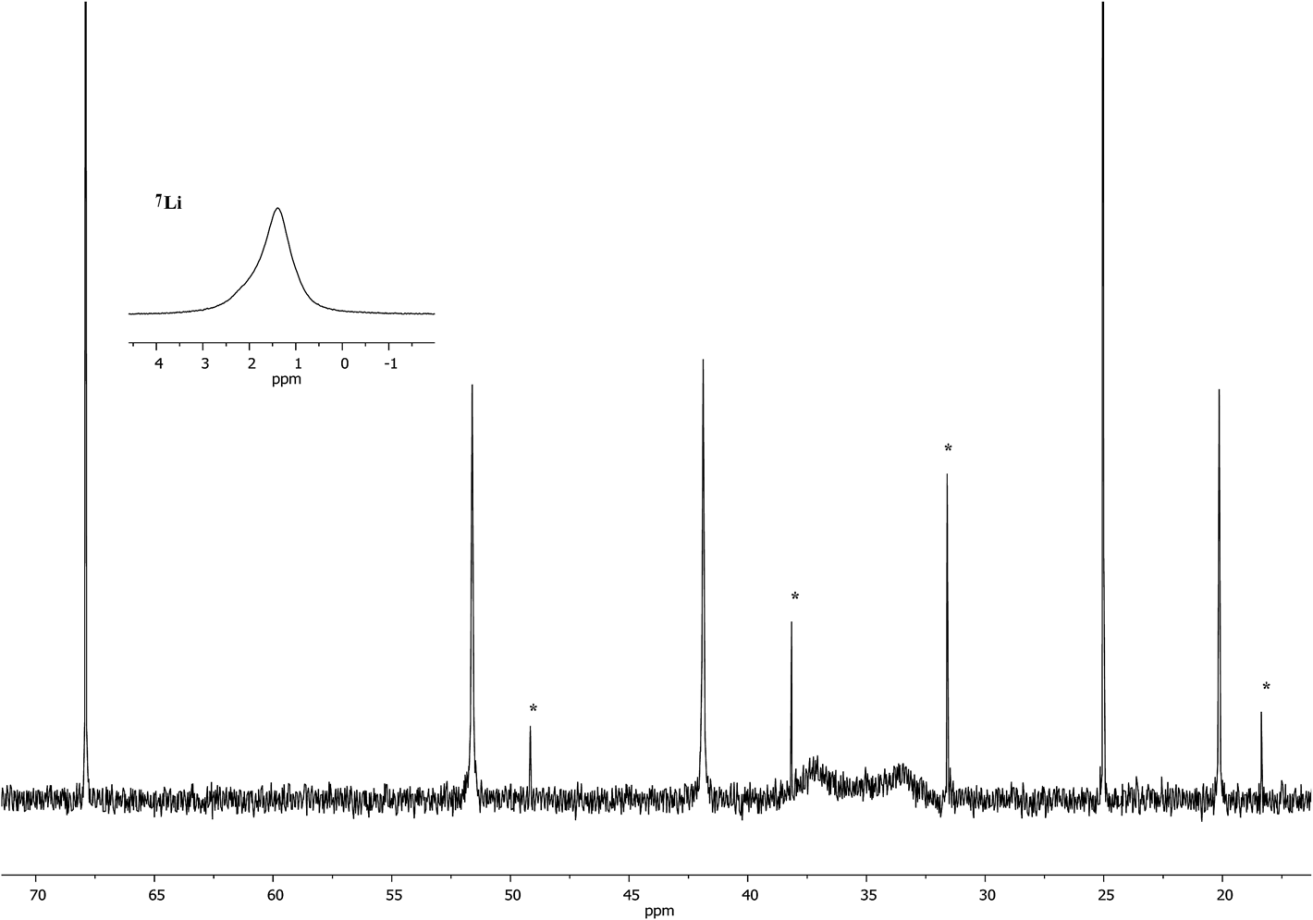
^13^C NMR spectrum of **11** in C_6_D_6_ (main); ^7^Li NMR spectrum (inset). *TMPH.

Interpretation of the convoluted solution behaviour for the systems described above has been attempted. It was anticipated that compound **8** would yield two species in solution – **8a** and **8b**. In the event, NMR spectroscopy of the bulk material could not be reconciled with this simple model of metal disorder. ^7^Li NMR spectroscopy, for example, revealed multiple solution species (Fig. 6). With NMR spectra of **9a** reported previously,^19^ its presence at as a minor component of this mixture at *δ* 0.90 ppm was easily established. To aid the assignment of other components, the spectroscopy of (TMP)_2_(OCN)Li_3_(THF)_2_ **11** was examined. The reproducible *in situ* reformation of a limited amount of TMPH in all TMP-based systems is attributed to the presence of trace moisture in the deuterated solvent in spite of its storage over molecular sieves (3 Å). This notwithstanding, ^13^C NMR spectroscopy revealed the existence of **11** as the only species observable in solution (Fig. 7).

**Fig. 8:**
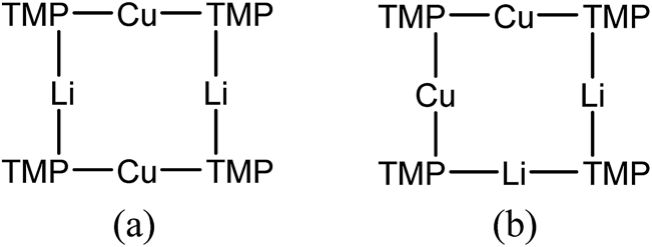
(a) The dimer of **9a** reveals one distinct TMP environment, whereas (b) dimers of **9b** and **9c** can aggregate to give ***i*-9a**, which reveals three.

Returning to the ^7^Li NMR spectrum of bulk **8**, the broad features at *δ* 2.18 and 1.41 ppm can now be accounted for by **11** and, by comparison with an authentically prepared sample (see ESI†), **9b**, respectively, leaving two signals to be identified at *δ* 1.64 ppm and at *δ* 0.48 ppm. To assign the first of these, ^1^H NMR spectroscopy was used. Firstly, THF notwithstanding, the ^1^H NMR spectrum of **11** was dominated by three singlets, at *δ* 1.76, 1.57 and 1.39 ppm in a 1 : 2 : 1 integral ratio. HSQC spectroscopy identified these as belonging to TMP-Me groups, and comparison with the TMP-Me resonances in authentic samples of **9a–c** (see ESI†) revealed that each of the aforementioned singlets falls within the expected chemical shift range for these compounds. Overall, these data suggest an isomeric variant of the previously reported dimer of Gilman cuprate **9a** (Fig. 8a).^19^ We propose inverse-**9a** (***i*-9a**); a tetranuclear metallacycle incorporating *adjacent pairs* of Li and Cu centres (Fig. 8b) best viewed as resulting from the agglomeration of dimers of TMPLi **9b** and TMPCu **9c**. The arrangement of ***i*-9a** yields three distinct TMP environments (Fig. 8). We hypothesise ***i*-9a** to be a kinetic product since it results formally from the catenation of dinuclear units of **9b** and **9c** *without requiring* the formation of Gilman cuprate **9a**. This thesis suggests that under suitable conditions, ***i*-9a** will rearrange to **9a**_2_. To test this, a typical sample of bulk **8** was heated to reflux and allowed to stand at room temperature. Fine needles crystallized, which were shown to be (thermodynamically preferred) Gilman cuprate by NMR spectroscopy.

Lastly, the minor signal at *δ* 0.48 ppm in Fig. 6 most logically belonged to either **8a** or **8b**. The spectroscopic analysis of pure **8a** was therefore carried out. This revealed a system whose behaviour paralleled that of previously reported **5a**,^21^ with the deposition of a fine white powder occurring upon sample dissolution in C_6_D_6_ (presumed LiOCN in this case). Except for the presence of THF, the signals matched those of **9a** (^7^Li NMR *δ* 0.90 ppm). This suggests that the signal at *δ* 0.48 ppm in Fig. 6 is due to **8b** not **8a**.

Both the metal disorder in single crystal **8** and the presence of multiple products in the bulk reaction mixture are unusual features in amidocuprate systems. However, they can be explained by reactions (1)–(3) below. In (1), CuOCN reacts with **9b** to expel LiOCN. This is rapidly sequestered in (2) at a rate competitive with (3). Remaining **9b** reacts with *in situ* formed **9c**(3) to then generate any compound in the series TMP_*m*+*n*_Cu_*m*_Li_*n*_ (*e.g.* ***i*-9a**). The failure to isolate ***i*-9a** in previous lithium amidocuprate work is best explained by a combination of the kinetic properties of ***i*-9a** and the ability of **9a** (but not ***i*-9a**) to also be generated by the dissociation of Lipshutz-type cuprates.

Formation of TMPCu **9c**
1
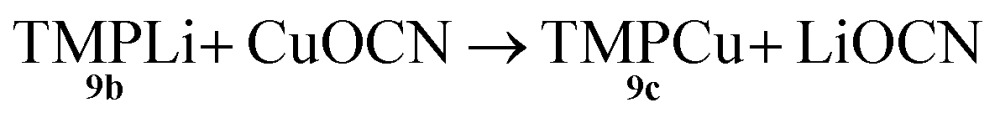



Formation of TMP_2_Li_3_OCN(THF)_2_ **11**
2
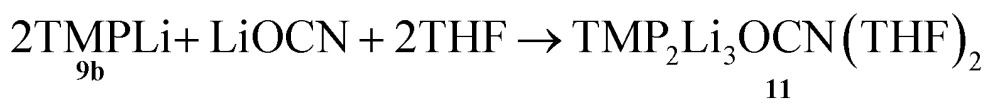



Formation of TMP_2_CuLi **9a** and ***i*-9a**
3
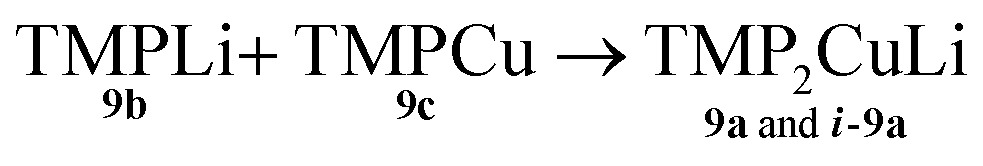



Moving to the behaviour of lithium amidocuprates in more polar reaction media, the 2 : 1 reaction of TMPLi and CuOCN in THF followed by recrystallization from hexane gave **9**, which crystallographic refinement indicated to be rich in Cu (Fig. 3), though the individual contributions of TMP_*m*+*n*_Cu_*m*_Li_*n*_ could not be established by solid state analysis. In contrast, ^7^Li NMR spectroscopy in C_6_D_6_ (see ESI†) identified three Li-containing species in solution: a minor signal at *δ* 1.65 ppm which matched ***i*-9a**, a dominant resonance at *δ* 0.91 ppm corresponding to **9a** and a shoulder at *δ* 0.94 ppm. Since **9a** and ***i*-9a** contain equal amounts of Cu and Li, it was anticipated that the unidentified species should be rich in Cu to ensure that the sample was Cu-rich overall. Strong contenders include TMP_3_Cu_2_Li and TMP_4_Cu_3_Li – since literature precedents exist for trimeric and tetrameric variants of **9b** ^36–38^ and **9c**.^39,40^

**Fig. 9:**
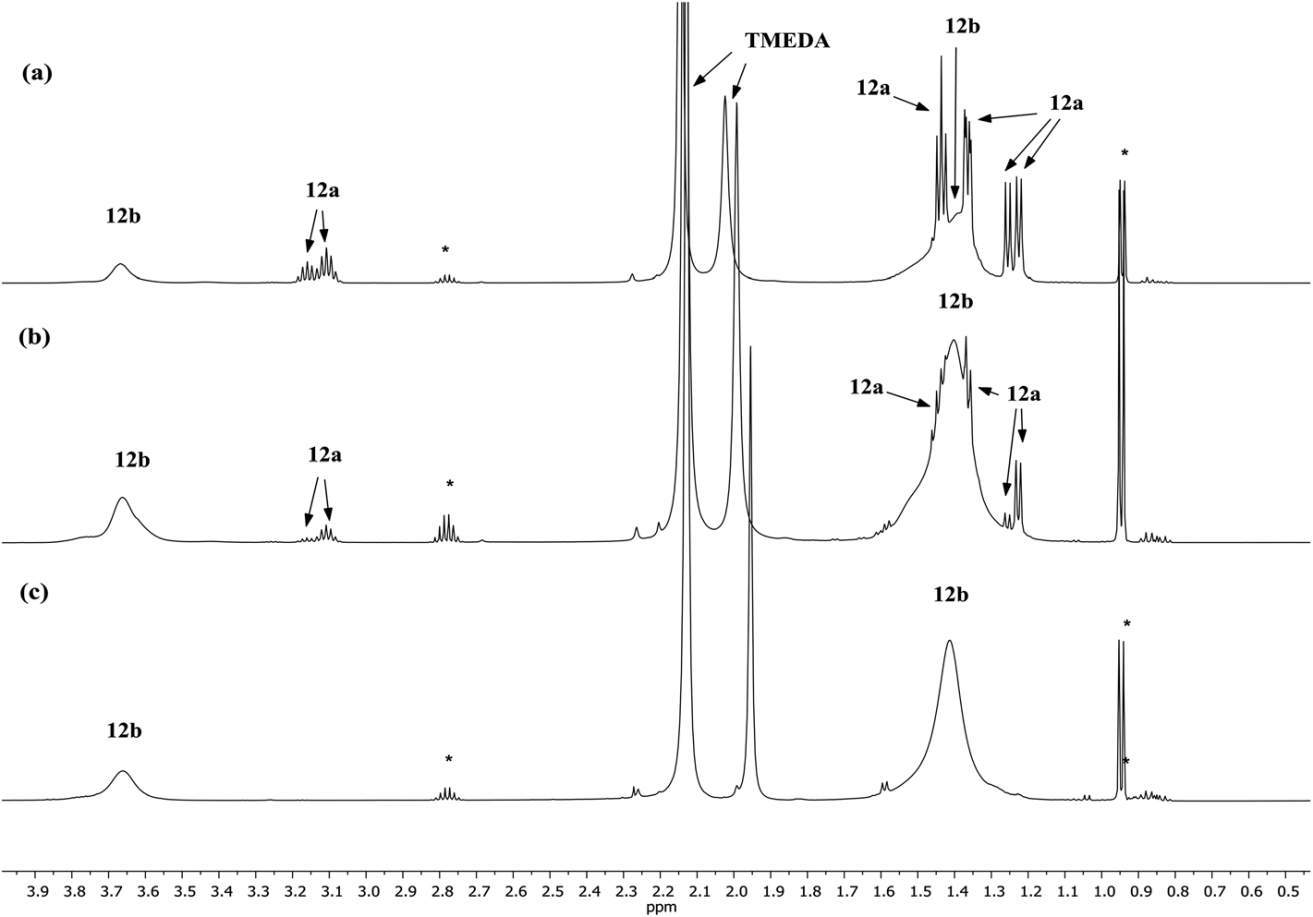
^1^H NMR spectra in C_6_D_6_ of (a) **12** (b) **12** (c) **12b**. *DAH.

Lastly, the solution behaviour of co-crystalline (DA)_2_Cu(Br)Li_2_(TMEDA)_2_ **12a**/(DA)_2_Li(Br)Li_2_(TMEDA)_2_ **12b** was probed in conjunction with that of pure **12b**. The data for pure **12b** proves relatively straightforward. It suggests one species dominant in solution (alongside minimal reformation of DAH,^41^ Fig. 9). Most clearly, ^7^Li NMR spectroscopy reveals signals in the expected 1 : 2 ratio at *δ* 2.28 and 1.26 ppm (ESI, Fig. S11c†). Moving to co-crystalline **12**, peaks corresponding to **12b** are added to by signals for **12a** (ESI, Fig. S12a–f†). ^1^H NMR spectroscopic data for multiple samples of **12** clearly show the levels of **12a** and **12b** to be variable (Fig. 9), pointing to the possibility of incorporating higher levels of Cu than evidenced by X-ray crystallography.

## Conclusions

The recent development of thiocyanatocuprate reagents has been extended to yield cyanatocuprates. Copper–lithium exchange in this new field has been evidenced by the observation of co-crystalline products of the type (TMP)_2_M(OCN)Li_2_ where M = Cu, Li. An analysis of the geometry at M reinforces the view, recently expressed for X = CN, halide, and SCN,^21,23^ that in (amido)_2_M(X)Li_2_ systems (X = inorganic anion) Cu prefers a linear, dicoordiante geometry (*viz.* **8**). This disinclination for higher order structure formation is emphasised when, for M = Li, a tendency for transannulation emerges and M becomes tricoordinate within the same structure-type (*viz.* **11**). These data are reinforced by attempts to fabricate monomeric Lipshutz-type cuprates, with Cu linear in (DA)_2_Cu(Br)Li_2_(TMEDA)_2_ **12a** and amidolithium-lithiocuprate adduct **13** and Li trigonal in (DA)_2_(Br)Li_3_(TMEDA)_2_ **12b**.

Solution studies have helped elucidate the complex chemistry on offer. In particular, insights into the behaviour of bulk **8** and also of **11** establish the *in situ* formation of Gilman cuprate **9a**. We show that this can form directly or through the rearrangement of a newly observed species (***i*-9a**) that we propose to form kinetically from the combination of amidolithium **9b** and amidocopper **9c**. In the case of **12**, results from crystallographic refinement could be combined with spectroscopic studies of **12b** to distinguish copper-containing Lisphutz-type monomers from their lithium-only congeners. Work is being initiated to deconvolute the complex solution chemistry of **13** and to establish the synthetic portfolio of cyantocuprates. This is focusing on enhancing atom efficiency in directed *ortho* cupration^19^ and halopyridine^21^ derivatization by combining Gilman dimer **9a**_2_ with substoichiometric LiOCN. The current work demonstrates, for the first time, the ability of an inorganic salt to combine with a dimeric Gilman cuprate to yield a Lipshutz-type dimer. Taking this together with previous work establishing Lipshutz-type dimers as a source of reactive Gilman *monomers* ^19^ alongside reformed lithium salt, we are now testing the non-stoichiometric deployment of LiOCN with a view to furthering recent interest in transferring main group polar organometallic chemistry to the catalytic regime.^7d,42^

## Experimental section

### General synthetic and analytical details

Reactions were carried out under dry nitrogen, using double manifold and glove-box methods. Solvents were distilled off sodium (toluene) or sodium–potassium amalgam (THF, hexane) immediately before use. 2,2,6,6-Tetramethylpiperidine (TMPH) was purchased from Alfa Aesar and stored over molecular sieves (4 Å). Other chemicals were used as received. *n*BuLi (1.6 M in hexanes) was purchased from Acros and used as received. For details of the syntheses of Ba(OCN)_2_ **6**,^32^ CuOCN **7** (ref. 32) and (TMPH_2_)OCN **10** see the ESI.† The syntheses of reference materials TMPLi **9b** and TMPCu **9c** were based on the literature^36,43^ and details are provided in the ESI.† IR spectra were collected on a Perkin Elmer Spectrum One FT IR spectrometer. The abbreviations used are: m = medium, s = strong. NMR data were collected on a Bruker Avance III HD 500 MHz Smart Probe FT NMR spectrometer (500.200 MHz for ^1^H, 125.775 MHz for ^13^C, 194.397 for ^7^Li). Spectra were obtained at 25 °C using deuterated solvent stored over molecular sieves (3 Å). For ^1^H and ^13^C, chemical shifts are internally referenced to deuterated solvent and calculated relative to TMS. For ^7^Li, an external reference was used (1 M LiCl in D_2_O). Chemical shifts are expressed in *δ* ppm. The following abbreviations are used: br = broad, m = multiplet, s = singlet, sh = shoulder.

### Crystallographic details

For details of data collections see Table 1. Crystals were transferred from the mother liquor to a drop of perfluoropolyether oil mounted upon a microscope slide under cold nitrogen gas.^44^ Suitable crystals were attached to the goniometer head *via* a MicroLoop™, which was then centred on the diffractometer. Data were collected on a Bruker D8 Quest (Cu-Kα, *λ* = 1.54184 Å), each equipped with an Oxford Cryosystems low-temperature device (*T* = 180(2) K). Structures were solved using SHELXT,^45^ with refinement, based on F^2^, by full-matrix least squares.^46^ Non-hydrogen atoms were refined anisotropically (for disorder, standard restraints and constraints were employed as appropriate) and a riding model with idealized geometry was employed for the refinement of H-atoms. Crystals of **8a** grew as two-component non-merohedral twins (see ESI†). Data have been deposited with the Cambridge Crystallographic Data Centre as supplementary publications CCDC ; 1540281 (**8**), ; 1540280 (**8a**), ; 1540286 (**9**), ; 1540282 (**11**), ; 1540284 (**12**), ; 1540285 (**12b**) and ; 1540283 (**13**).

**Table 1:** X-ray crystal data for **8**_2_, **8a**_2_, **9**, **11**_2_, **12**, **12b** and **13**

	**8**_2_	**8a**_2_	**9**	**11**_2_	**12**	**12b**	**13**
Formula	C_46_H_88_Cu_0.21_Li_5.79_N_6_O_4_	C_46_H_88_Cu_2_Li_4_N_6_O_4_	C_36_H_72_Cu_2.7_Li_1.3_N_4_	C_54_H_104_Li_6_N_6_O_6_	C_24_H_60_BrCu_0.09_Li_2.91_N_6_	C_24_H_60_BrLi_3_N_6_	C_37_H_88_CuLi_4_N_9_O
*M*	842.74	944.06	741.41	975.07	538.60	533.51	766.46
Crystal system	Triclinic	Triclinic	Monoclinic	Triclinic	Monoclinic	Monoclinic	Triclinic
Space group	*P*1	*P*1	*P*2_1_/*c*	*P*1	*P*2_1_/*c*	*P*2_1_/*c*	*P*1
*a*	7.9156(3)	8.4118(4)	11.6661(3)	11.8638(3)	17.7591(8)	17.7718(4)	11.9447(8)
*b*	13.4775(5)	11.5807(5)	22.7624(5)	12.0430(3)	12.2389(6)	12.1985(3)	13.7635(9)
*c*	13.8111(6)	13.5645(6)	15.2664(4)	12.2033(3)	17.2889(8)	17.2945(5)	16.4214(11)
*α*	110.986(2)	98.376(2)	90	82.4990(10)	90	90	80.989(3)
*β*	94.546(2)	95.058(2)	108.6770(10)	66.9060(10)	118.227(2)	118.2370(10)	78.164(3)
*γ*	104.331(2)	94.955(2)	90	72.4890(10)	90	90	67.824(3)
*V*	1309.70(9)	1295.58(10)	3840.48(17)	1529.35(7)	3310.9(3)	3303.09(15)	2437.5(3)
*Z*	1	1	4	1	4	4	2
*ρ*_calcd_	1.068	1.210	1.282	1.059	1.081	1.073	1.044
*μ*	0.583	1.344	1.952	0.511	1.878	1.823	0.881
Data	17 570	4430	19 840	16 485	25 251	26 193	35 010
Unique data	4631	4430	5479	5374	5837	5811	8630
*R*_int_	0.0395	0.0361^*a*^	0.0469	0.0303	0.0843	0.0562	0.0420
*θ* (°)	3.488–66.840	3.311–66.793	3.621–59.106	3.849–66.691	2.824–66.842	2.822–66.687	2.760–66.920
w*R*_2_	0.1388	0.1026	0.1167	0.1400	0.1524	0.0901	0.1881
*R*	0.0515	0.0351	0.0501	0.0502	0.0825	0.0414	0.0572
GoF	1.026	1.082	1.055	1.035	1.117	1.023	1.048
Parameters	298	289	417	343	317	307	513
Peak/hole (eÅ^–3^)	0.431/–0.450	0.261/–0.452	0.787/–0.382	0.535/–0.313	0.510/–0.632	0.277/–0.345	0.547/–0.874

^*a*^Based on agreement between observed single and composite intensities and those calculated from refined unique intensities and twin fractions.

### Synthesis and characterization of (TMP)_2_Cu_0.1_Li_0.9_(OCN)Li_2_(THF) **8**

To a stirred solution of TMPH (0.34 mL, 2 mmol) and THF (0.08 mL, 1 mmol) in hexane (4 mL) was added *n*BuLi (1.25 mL, 1.6 M in hexanes, 2 mmol) at –78 °C. The mixture was returned to room temperature to give a yellow solution. This was added to a suspension of CuOCN (0.11 g, 1 mmol) in hexane (1 mL) at –78 °C. The mixture was returned to room temperature to give a pale yellow suspension. Filtration gave a yellow solution and storage at –27 °C gave crystalline material from which a well-faceted, block-like crystal of (TMP)_2_Cu_0.1_Li_0.9_(OCN)Li_2_(THF) was selected for X-ray diffraction. The following characterization refers to the *bulk* crystalline product. Yield 54 mg, melting point dec. from 125 °C. Selected IR spectroscopy (nujol) *ν̄* 2208 (s, CN), 1340 (m, CO), 1229 (m, CO) cm^–1^. ^1^H NMR spectroscopy (500 MHz, C_6_D_6_) *δ* 3.56 (m, 8H, THF), 2.17–1.77 (br, 8H, TMP-4), 1.75 (s, 6H, TMP-Me), 1.74–1.60 (br, m, 12H, TMP-3,5, TMP-Me), 1.57 (s, 12H, TMP-Me), 1.56–1.41 (br, m, 12H, TMP-3,5, TMP-4, TMP-Me), 1.39 (s, 6H, TMP-Me), 1.36 (m, 8H, THF), 1.34–1.13 (br, 12H, TMP-3,5), 1.12–1.07 (m, 4H, TMP-3,5), 1.06 (s, 2H, TMPH-Me). ^13^C NMR (125 MHz, C_6_D_6_) *δ* 64.8 (THF), 56.9 (TMP-2,6 ***i*-9a**), 54.2 (TMP-2,6 ***i*-9a**/TMP-2,6 **9a**), 53.4 (TMP-2,6 **8**), 52.0 (TMP-2,6 ***i*-9a**), 51.6 (TMP-2,6 **11**), 49.2 (TMPH-2,6), 42.6 (TMP-3,5 ***i*-9a**), 42.5 (TMP-3,5 ***i*-9a**), 42.4 (TMP-3,5 **8**), 42.1 (TMP-3,5 ***i*-9a**/TMP-3,5 **9a**), 41.9 (TMP-3,5 **11**), 40.5 (TMP-Me **8**), 40.2 (TMP-Me **9a**), 39.7 (TMP-Me ***i*-9a**), 38.1 (TMPH-3,5), 37.5 (br, TMP-Me **11**), 37.0 (TMP-Me ***i*-9a**), 36.8 (TMP-Me ***i*-9a**), 35.7 (TMP-Me **8**), 34.5 (TMP-Me **9a**), 34.2 (TMP-Me ***i*-9a**), 33.5 (br, TMP-Me **11**), 31.6 (TMPH-Me), 25.1 (THF), 20.1 (TMP-4 **11**), 19.6 (TMP-4 ***i*-9a**), 19.2 (TMP-4 ***i*-9a**/TMP-4 **9a**), 19.1 (TMP-4 **8**), 19.1 (TMP-4 ***i*-9a**), 18.4 (TMPH-4). ^7^Li NMR (194 MHz, C_6_D_6_) *δ* 2.17 (br, s, 1Li, **9b**), 1.64 (s, 1.5Li, ***i*-9a**), 1.39 (br, s, 2Li, **11**), 0.90 (s, 0.5Li, **9a**), 0.48 (s, 0.5Li, **8**).

### Synthesis and characterization of (TMP)_2_Cu(OCN)Li_2_(THF) **8a**

To a stirred solution of TMPH (0.68 mL, 4 mmol) in hexane (2 mL) and toluene (2 mL) was added *n*BuLi (2.5 mL, 1.6 M in hexanes, 4 mmol) at –78 °C. The solution was warmed to room temperature and transferred to a suspension of CuSCN (0.243 g, 2 mmol) in hexane (2 mL) and toluene (2 mL). The suspension was warmed to room temperature then heated to reflux whereupon a grey discolouration was observed. To remove LiSCN, this mixture was filtered whilst hot onto LiOCN (0.10 g, 2 mmol) and the solvent was removed *in vacuo*. THF (6 mL) was added and the suspension was stirred at room temperature for 30 minutes, during which time partial dissolution of the LiOCN occurred. The THF was removed *in vacuo* to give a sticky solid, which dissolved when hexane (6 mL) was added. The solution was filtered and the filtrate stored at –27 °C for 1 week during which time **8a** deposited as radiating fans of crystals. Yield 310 mg (33% wrt. CuSCN), melting point 192–194 °C. Elemental analysis C_24_H_48_CuLi_2_N_3_O_2_, requires (%) C, 58.52; H, 9.40; N, 8.90. Found (%) C, 58.06; H, 9.38; N, 9.01. Selected IR spectroscopy (nujol) *ν̄* 2241 (s, CN), 2208 (s, CN), 1340 (m, CO), 1228 (m, CO) cm^–1^. ^1^H NMR spectroscopy (500 MHz, C_6_D_6_) *δ* 3.57 (br, m, 4H, THF), 1.89–1.76 (br, m, 2H, TMP-4), 1.67–1.61 (m, 4H, TMP-3,5), 1.60 (s, 12H, TMP-Me), 1.59–157 (m, 2H, TMP-4), 1.56 (s, 12H, TMP-Me), 1.41 (br, m, 4H, THF), 1.09 (m, 4H, TMP-3,5), 1.06 (s, 1.3H, TMPH-Me). ^13^C NMR spectroscopy (125 MHz, C_6_D_6_) *δ* 67.4 (br, THF), 54.2 (TMP-2,6), 49.2 (TMPH-2,6), 42.1 (TMP-3,5), 40.1 (TMP-Me), 38.2 (TMPH-3,5), 34.5 (TMP-Me), 31.6 (TMPH-Me), 25.3 (br, THF), 19.2 (TMP-4), 18.4 (TMPH-4). ^7^Li NMR spectroscopy (194 MHz, C_6_D_6_) *δ* 0.90.

### Synthesis and characterization of (TMP)_2_Cu_1.35_Li_0.65_ **9**

To a stirred solution of TMPH (0.34 mL, 2 mmol) and THF (2 mL) was added *n*BuLi (1.25 mL, 1.6 M in hexanes, 2 mmol) at –78 °C. The mixture was left to reach room temperature. The resulting yellow solution was transferred to a suspension of CuOCN (0.11 g, 1 mmol) in THF (1 mL) at –78 °C. The mixture was returned to room temperature to give a pale yellow suspension. The solvent was removed and the resulting yellow solid dissolved in hexane (4 mL). Filtration gave a yellow solution that was stored at –27 °C to give a crystalline aggregate which analysed as (TMP)_2_Cu_1.35_Li_0.65_ by X-ray diffraction. Yield 65 mg. ^1^H NMR spectroscopy (500 MHz, 298 K, C_6_D_6_) *δ* 1.89–1.80 (m, 4H, TMP-4), 1.79 (s, 6H, TMP-Me), 1.76 (s, 8H, TMP-Me), 1.73 (s, br, 6H, TMP-Me), 1.72–1.69 (br, m, 4H, TMP-4), 1.68–1.61 (m, 4H, TMP-3,5), 1.59 (s, 12H, TMP-Me; TMP-Me **9a**), 1.57 (s, 10H, TMP-Me; TMP-3,5; TMP-4), 1.56 (s, 12H, TMP-Me **9a**), 1.10 (m, 6H, TMP-3,5), 1.06 (s, 1H, TMPH-Me).^13^C NMR spectroscopy (125 MHz, 298 K, C_6_D_6_) *δ* 56.9 (TMP-2,6), 54.2 (TMP-2,6), 54.2 (TMP-2,6 **9a**), 49.2 (TMPH-2,6), 42.6 (TMP-3,5), 42.5 (TMP-3,5), 42.1 (TMP-3,5 **9a**), 42.1 (TMP-3,5), 40.1 (TMP-Me **9a**), 39.7 (TMP-Me), 38.2 (TMPH-3,5), 37.6 (br, TMP-Me), 37.2 (br, TMP-Me), 36.6 (br, TMP-Me), 34.8 (TMP-Me), 34.5 (TMP-Me **9a**), 31.6 (TMPH-Me), 19.3 (TMP-4), 19.2 (TMP-4 **9a**), 19.2 (TMP-4), 18.4 (TMPH-4).^7^Li NMR spectroscopy (194 MHz, 298 K, C_6_D_6_) *δ* 1.65 (s, 0.16Li), 0.94 (sh, 0.8Li), 0.91 (s, 1.0Li, **9a**).

### Synthesis and characterization of (TMP)_2_(OCN)Li_3_(THF)_2_ **11**

#### Method (a)

A suspension of (TMPH_2_)OCN **10** (0.09 g, 0.5 mmol) in THF (2 mL) was treated with TMPH (0.08 mL, 0.5 mmol). *n*BuLi (0.95 mL, 1.5 mmol) was added dropwise at –78 °C and the resulting suspension was left to warm to room temperature, whereupon it dissolved. The solvent was removed *in vacuo* and replaced with hexane (3 mL). The colourless solution was filtered, with storage of the filtrate at –27 °C for 1 day giving colourless crystals. Yield 65 mg (27% wrt. NCO), melting point 90–92 °C. Selected IR spectroscopy (nujol) *ν̄* 2207 (s, CN), 1352 (s, CO), 1226 (s, CO) cm^–1^. ^1^H NMR spectroscopy (500 MHz, C_6_D_6_) *δ* 3.56 (m, 8H, THF), 2.27–1.37 (br, m, 30H, TMP-3,4,5,Me), 1.35 (s, 6H, TMP-Me), 1.34 (m, 8H, THF), 1.06 (s, 3.2H, TMPH-Me), 0.31 (br, s, 0.31H, TMPH-NH). ^13^C NMR (126 MHz, C_6_D_6_) *δ* 67.8 (THF), 52.0 (TMP-2,6 **9b**), 51.7 (TMP-2,6 **11**), 49.2 (TMPH-2,6), 42.4 (TMP-3,5 **9b**), 41.9 (TMP-3,5 **11**), 38.2 (TMPH-3,5), 37.5 (br, TMP-Me **11**), 36.5 (TMP-Me **9b**), 33.5 (br, TMP-Me **11**), 31.6 (TMPH-Me), 25.0 (THF), 20.2 (TMP-4 **11**), 18.4 (TMPH-4). ^7^Li NMR (194 MHz, C_6_D_6_) *δ* 2.21 (s, br, 1Li, **9b**), 1.48 (s, br, 2Li, **11**), –1.54 (s, 0.2Li, unidentified).

#### Method (b)

TMPH (0.34 mL, 2 mmol) in THF (2 mL) was treated with *n*BuLi (1.25 mL, 1.6 M in hexanes, 2 mmol) at –78 °C. The pale yellow solution was returned to room temperature whereupon it was transferred to a suspension of LiOCN (0.05 g, 1 mmol) in THF at –78 °C. The mixture warmed to room temperature and was stirred for *ca.* 15 minutes, during which time the LiOCN was observed to dissolve. The solvent was removed *in vacuo*, leaving a sticky white solid which dissolved upon the addition of hexane (6 mL) with gentle warming. The solution was filtered and the filtrate stored at –27 °C for 24 h hours to give colourless crystals. Yield 75 mg (15% wrt LiOCN). Elemental analysis, C_27_H_52_Li_3_N_3_O_3_ requires (%) C 66.52, H 10.75, N 8.62; found (%) C, 66.08; H, 10.76; N, 8.83. ^1^H NMR (500 MHz, C_6_D_6_): *δ* 3.56 (m, 8H, THF), 2.31–1.38 (br, 30H, TMP), 1.35 (m, 8H, THF), 1.32–1.12 (br, 6H, TMP), 1.06 (s, 1H, TMPH). ^13^C NMR (125 MHz, C_6_D_6_): *δ* 67.9 (THF), 51.6 (TMP-2,6), 49.2 (TMPH-2,6), 41.9 (TMP-3,5), 38.2 (TMPH-3,5), 37.1 (br, TMP-Me), 33.5 (br, TMP-Me), 31.6 (TMPH-Me), 25.0 (THF), 20.1 (TMP-4), 18.4 (TMPH-4). ^7^Li NMR (194 MHz, C_6_D_6_): *δ* 1.38 (s, br).

### Synthesis and characterization of (DA)_2_Cu_0.09_Li_0.91_(Br)Li_2_(TMEDA)_2_ **12**

*n*BuLi (2.5 mL, 1.6 M in hexanes, 4 mmol) was added to a stirred solution of DAH (0.56 mL, 4 mmol) and TMEDA (0.6 mL, 4 mmol) in hexane (4 mL) at –78 °C. The resulting solution was returned to room temperature to give a yellow solution that was transferred to a –78 °C suspension of CuBr (0.28 g, 2 mmol) in hexane (2 mL). The mixture was returned to room temperature to give a brown suspension. Filtration gave a pale yellow solution. Storage of this at –27 °C for 24 hours gave colourless blocks of cocrystalline Lipshutz-type (DA)_2_Cu(Br)Li_2_(TMEDA)_2_ **12b** and (DA)_2_(Br)Li_3_(TMEDA)_2_ **12a**. Yield 350 mg.

#### Representative sample (**1**)

^1^H NMR spectroscopy (500 MHz, C_6_D_6_) *δ* 3.76 (sh, br, 0.36H, DA–CH, **12a**), 3.67 (s, br, 3.15H, DA–CH, **12b**), 3.25 (septet, ^3^ *J*_HH_ = 6 Hz, 0.02H, DA–CH, **12a**), 3.16 (septet, ^3^ *J*_HH_ = 6 Hz, 0.08H, DA–CH, **12a**), 3.10 (septet, ^3^ *J*_HH_ = 6 Hz, 0.28H, DA–CH, **12a**), 2.78 (octet, ^3^ *J*_HH_ = 6 Hz, 0.4H, DAH–CH), 2.13 (s, 24H, TMEDA-Me), 1.99 (s, 8H, TMEDA–CH_2_), 1.63–1.20 (br, m, 24H, **12a** + **12b**), 0.95 (d, ^3^ *J*_HH_ = 6 Hz, 2H, DAH–Me). ^13^C NMR (126 MHz, C_6_D_6_) *δ* 57.0 (TMEDA–CH_2_), 50.1 (DA–CH, **12a**), 48.8 (DA–CH, **12b**), 46.5 (TMEDA–CH_3_), 44.9 (DAH–CH), 28.2, 27.0 (DA–Me, **12a**), 26.2 (br, DA–Me, **12b**), 23.2 (DAH–Me). ^7^Li NMR (194 MHz, C_6_D_6_) *δ* 2.34 (s, 1Li, (DA)_2_Li, **12b**), 1.58 (s, 0.4Li, Li(TMEDA), **12a**), 1.18 (s, 2Li, Li(TMEDA), **12b**).

#### Representative sample (**2**)

^1^H NMR spectroscopy (500 MHz, C_6_D_6_) *δ* 3.76 (sh, br, 0.11H, DA–CH, **12a**), 3.67 (s, br, 1.90H, DA–CH, **12b**), 3.25 (septet, ^3^ *J*_HH_ = 6 Hz, 0.02H, DA–CH, **12a**), 3.16 (septet, ^3^ *J*_HH_ = 6 Hz, 0.64H, DA–CH, **12a**), 3.11 (septet, ^3^ *J*_HH_ = 6 Hz, 1.00H, DA–CH, **12a**), 2.78 (octet, ^3^ *J*_HH_ = 6 Hz, 0.16H, DAH–CH), 2.14 (s, 24H, TMEDA–Me), 2.02 (s, 8H, TMEDA–CH_2_), 1.60–1.31(br, m, 20H, DA–Me, **12a** + **12b**), 1.25 (d, ^3^ *J*_HH_ = 6 Hz, 2H, **12a**), 1.22 (d, ^3^ *J*_HH_ = 6 Hz, 2H, **12a**), 0.94 (d, ^3^ *J*_HH_ = 6 Hz, 1H, DAH–Me). ^13^C NMR (125 MHz, C_6_D_6_) *δ* 57.0 (TMEDA–CH_2_), 50.9 (DA–CH, **12a**), 50.4 (DA–CH, **12a**), 50.1 (DA–CH, **12a**), 48.8 (DA–CH, **12b**), 46.3 (TMEDA–Me), 44.8 (DAH–CH), 28.4 (DA–Me, **12a**), 28.2 (DA–Me, **12a**), 27.6 (DA–Me, **12a**), 27.5 (DA–Me, **12a**), 27.0 (DA–Me, **12a**), 26.9 (DA–Me, **12a**), 26.1 (br, DA–Me, **12b**), 23.4 (DAH–Me). ^7^Li NMR (194 MHz, C_6_D_6_) *δ* 2.37 (s, 1.0Li, **12b**), 1.63 (s, 1.2Li, **12a**), 1.59 (s, 1.0Li, **12a**), 1.17 (s, 2Li, **12b**).

### Synthesis and characterization of (DA)_2_(Br)Li_3_(TMEDA)_2_ **12b**

To a suspension of DAH·HBr (0.18 g, 1 mmol) in hexane (6 mL) was added DAH (0.14 mL, 1 mmol) and TMEDA (0.30 mL, 2 mmol). The mixture was cooled to –78 °C, treated with *n*BuLi (1.9 mL, 1.6 M in hexanes, 3 mmol) and returned to room temperature to give a pale yellow solution. The solution was filtered, concentrated (to *ca.* 4 mL) and stored at –27 °C for 1 day after which colourless block-like crystals of **12b** were deposited. Yield 320 mg (60% wrt. Br), melting point 76–78 °C. Elemental analysis, C_24_H_60_BrLi_3_N_6_ requires (%) C 54.03, H 11.34, N 15.75; found (%) C 54.09, H 11.66, N 15.58. ^1^H NMR spectroscopy (500 MHz, C_6_D_6_) *δ* 3.66 (s, br, 2H, DA–CH), 2.79 (octet, ^3^ *J*_HH_ = 6 Hz, 0.06H, DAH–CH), 2.13 (s, 24H, TMEDA–Me), 1.96 (s, 8H, TMEDA–CH_2_), 1.41 (s, br, 24H, DA–Me), 0.95 (d, ^3^ *J*_HH_ = 6 Hz, 1.35H, DAH–Me). ^13^C NMR (126 MHz, C_6_D_6_) *δ* 56.9 (TMEDA–CH_2_), 48.8 (DA–CH), 46.5 (TMEDA–CH_3_), 44.9 (DAH–CH), 26.2 (DA–Me), 23.2 (DAH–Me). ^7^Li NMR (194 MHz, C_6_D_6_) *δ* 2.28 (s, br, 1Li, (DA)_2_Li), 1.26 (s, 2Li, Li(TMEDA)).

### Synthesis and characterization of (DA)_4_Cu(OCN)Li_4_(TMEDA)_2_ **13**

*n*BuLi (1.25 mL, 1.6 M in hexanes, 2 mmol) was added to a stirred solution of DAH (0.28 mL, 2 mmol) and TMEDA (0.3 mL, 2 mmol) in hexane (4 mL) at –78 °C. The resulting solution was returned to room temperature to give a yellow solution that was transferred to a suspension of CuOCN (0.11 g, 1 mmol) in hexane (1 mL) at –78 °C. The mixture was returned to room temperature to give a grey suspension, which was filtered to give a pale yellow solution. Storage at +5 °C for 24 hours gave white needle-like crystals of **13**. Yield 0.11 g (14% wrt. CuOCN), melting point dec. *ca.* 95 °C. Elemental analysis, C_37_H_88_CuLi_4_N_9_O requires (%) C, 57.98; H, 11.57; N, 16.45. Found: C, 57.77; H, 11.68; N, 16.76. Selected IR spectroscopy (nujol) *ν̄* 2207 (s, CN), 1353 (m, CO), 1293 (m, CO) cm^–1^. ^1^H NMR spectroscopy (500 MHz, C_6_D_6_) *δ* 3.88–3.40 (br, m, 6.2H, DA–CH), 3.28 (br, m, 0.53H, DA–CH), 3.14 (m, 0.15H, DA–CH), 3.11 (septet, ^3^ *J*_HH_ = 6 Hz, 1.12H, DA–CH), 2.78 (octet, ^3^ *J*_HH_ = 6 Hz, 0.14H, DAH–CH), 2.01 (s, 24H, TMEDA–Me), 1.94 (s, 8H, TMEDA–CH_2_), 1.71–1.38 (br, m, 22H, DA–Me), 1.37 (d, ^3^ *J*_HH_ = 6 Hz, 3H, DA–Me), 1.35–1.25 (br, m, 16H, DA–Me), 1.23 (d, ^3^ *J*_HH_ = 6 Hz, 3H, DA–Me), 1.22–1.14 (br, m, 4H, DA–Me), 0.94 (d, ^3^ *J*_HH_ = 6 Hz, 0.8H, DAH–Me). ^13^C NMR (125 MHz, C_6_D_6_) *δ* 57.3 (TMEDA–CH_2_), 50.1 (DA–CH), 49.8 (DA–CH), 49.6 (DA–CH), 49.2 (DA–CH), 48.6 (DA–CH), 48.3 (DA–CH), 45.9 (TMEDA–Me), 44.9 (DAH–CH), 44.8 (DAH–CH), 28.2 (DA–Me), 27.8 (DA–Me), 27.7 (DA–Me), 27.0 (DA–Me), 26.0 (DA–Me), 25.8 (DA–Me), 25.2 (DA–Me), 25.1 (DA–Me), 23.2 (DAH–Me), 23.1 (DAH–Me). ^7^Li NMR (194 MHz, C_6_D_6_) *δ* 2.34 (br, s, 0.78Li), 2.14 (sh, 0.22Li), 1.58 (s, 0.94Li), 0.84 (s, 0.40Li), 0.37 (s, 1.66Li).
