# Supplementary material for: Metal exchange in lithiocuprates: implications for our understanding of structure and reactivity
Source: Chem Sci. 2017 May 4;8(7):4904–16. doi: 10.1039/c7sc01423f (PMC5603898; doi:10.1039/c7sc01423f)
Supplement: Supplementary file 1 [file SC-008-C7SC01423F-s001.pdf]

Electronic Supplementary Material (ESI) for Chemical Science.
This journal is © The Royal Society of Chemistry 2017

Electronic Supplementary data

Metal exchange in lithiocuprates: implications for our understanding of structure and reactivity

Andrew J. Peel, Ryan Ackroyd and Andrew E. H. Wheatley*

Department of Chemistry, University of Cambridge, Lensfield Road, Cambridge, CB2 1EW (UK); Fax: (+) 44 1223 336362; e-mail: aehw2@cam.ac.uk

Synthesis and characterization of Ba(OCN)$_2$ 6 .... ***3***
Synthesis and characterization of CuOCN 7 .... ***3***
Figure S1 *Edge-on representation of the lithium-only component of **8$_2$***.............. ***4***
Additional characterisation for bulk product 8 .... ***4***
Figure S2a ^{1}H NMR spectrum 4
Figure S2b ^{13}C NMR spectrum 5
Figure S2c ^{7}Li NMR spectrum 5
Figure S3 *Molecular structure of pure **8a$_2$***.. ***6***
Additional characterization of pure (TMP)$_2$Cu(OCN)Li$_2$(THF) 8a ***6***
Figure S4a ^{1}H NMR spectrum 7
Figure S4b ^{13}C NMR spectrum 7
Figure S4c ^{7}Li NMR spectrum 8
Additional characterization of 9 .... ***8***
Figure S5a ^{1}H NMR spectrum 8
Figure S5b ^{13}C NMR spectrum 9
Figure S5c ^{7}Li NMR spectrum 9
Additional characterization of TMP$_2$CuLi 9a ***10***
Figure S6a ^{1}H NMR spectrum 10
Figure S6b ^{13}C NMR spectrum 10
Figure S6c ^{7}Li NMR spectrum 11
Synthesis and characterization of TMPLi 9b ***11***
Figure S7a ^{1}H NMR spectrum 12
Figure S7b ^{13}C NMR spectrum 12
Figure S7c ^{7}Li NMR spectrum 13

***Synthesis and characterization of TMPCu 9c*……………………………………..14**
Figure S8a ^{1}H NMR spectrum 14
Figure S8b ^{13}C NMR spectrum 15
***Synthesis and characterization of (TMPH$_2$)OCN 10*…………………………....15**
Figure S9a ^{1}H NMR spectrum 16
Figure S9b ^{13}C NMR spectrum 16
***Additional characterization of (TMP)$_2$(OCN)Li$_3$(THF)$_2$ 11*……………………17**
Figure S10a ^{1}H NMR spectrum 17
Figure S10b ^{13}C NMR spectrum 17
Figure S10c ^{7}Li NMR spectrum 18
Figure S4 *Molecular structure of pure* ***12b*...18**
***Additional characterization of (DA)$_2$CuBrLi$_2$(TMEDA)$_2$ 12b*………………… 19**
Figure S11a ^{1}H NMR spectrum 19
Figure S11b ^{13}C NMR spectrum 19
Figure S11c ^{7}Li NMR spectrum 20
***Additional characterization of (DA)$_2$Cu$_{0.09}$Li$_{0.91}$BrLi$_2$(TMEDA)$_2$ 12*………… 20**
Figure S12a ^{1}H NMR spectrum (sample 1) 20
Figure S12b ^{13}C NMR spectrum (sample 1) 21
Figure S12c ^{7}Li NMR spectrum (sample 1) 21
Figure S12d ^{1}H NMR spectrum (sample 2) 22
Figure S12e ^{13}C NMR spectrum (sample 2) 22
Figure S12f ^{7}Li NMR spectrum (sample 2) 23
***Additional characterization of (DA)$_4$Cu(OCN)Li$_4$(TMEDA)$_2$ 13*……………….23**
Figure S13a ^{1}H NMR spectrum 23
Figure S13b ^{13}C NMR spectrum 24
Figure S13c ^{7}Li NMR spectrum 24

Synthesis and characterization of Ba(OCN)$_2$ 6[1]

NaOCN (13 g, 0.2 mol) was dissolved in H_2O (115 mL). To the stirred solution $Ba(ClO_4)_2$ (0.5 g, 0.9 mmol) was added and after 15 min the precipitate was removed by filtration. Further $Ba(ClO_4)_2$ (33.4 g, 0.1 mol) was dissolved in methanol (115 mL) and added, with the resulting solution stirred for 1 h. The product was collected under suction, washed with methanol and dried in *vacuo* to give a white powder. Yield 11.08 g (50 %), melting point >300 °C. Elemental Analysis, $BaC_2N_2O_2$ requires (%) C 10.85, N 12.65; found (%) C 10.76, N 12.40. Selected IR spectroscopy (nujol) $\bar{\nu}$ 2188 (s, CN), 2155 (s, CN), 1320 (s, CO), 1298 (s, CO), 1227 (s, CO), 1210 (s, CO) cm^{-1}. ^{13}C NMR (125 MHz, D_2O) δ 128.7.

Synthesis and characterization of CuOCN 7[1]

$Cu(NO_3)_2(H_2O)_3$ (2.4 g, 0.01 mol) was added to a solution of $LiOAc(H_2O)_2$ (3 g, 0.03 mol) in water (8 mL) to give a dark blue solution. To this was added a solution of filtered $Li_2SO_4(H_2O)$ (1.28 g, 0.01 mol) and **6** (2.2 g, 0.01 mol) in H_2O (30 mL). Aqueous SO_2 (15 mL, 0.7 M) was added to the mixture until it became green. The resultant green solution was left for 30 mins, the precipitate collected by filtration, washed with deaerated water (2 mL) and dried *in vacuo*. Yield 0.54 g (51 %), melting point dec. >140 °C. Elemental Analysis, CuCNO requires (%) C 11.38, N 13.27; found (%) C 11.38, N 13.27. Selected IR spectroscopy (nujol) $\bar{\nu}$ 2116 (s) cm^{-1}. ^{13}C NMR (125 MHz, CD_3CN) δ 126.3 (t, J = 23 Hz) ppm.

---

1. E. Söderbäck, *Acta Chem. Scand.*, 1957, **11**, 1622-1634.

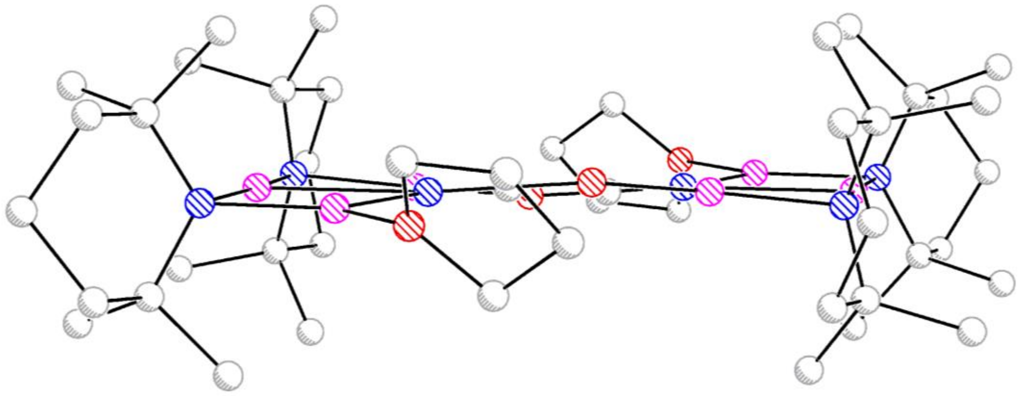

Figure S1 Edge-on representation of the lithium-only component of $\mathbf{8}_2$ (*i.e.* $\mathbf{8b}_2$), revealing the essentially planar character of the metallacyclic core and emphasizing the positions of the THF molecules.

Additional characterisation for bulk product 8

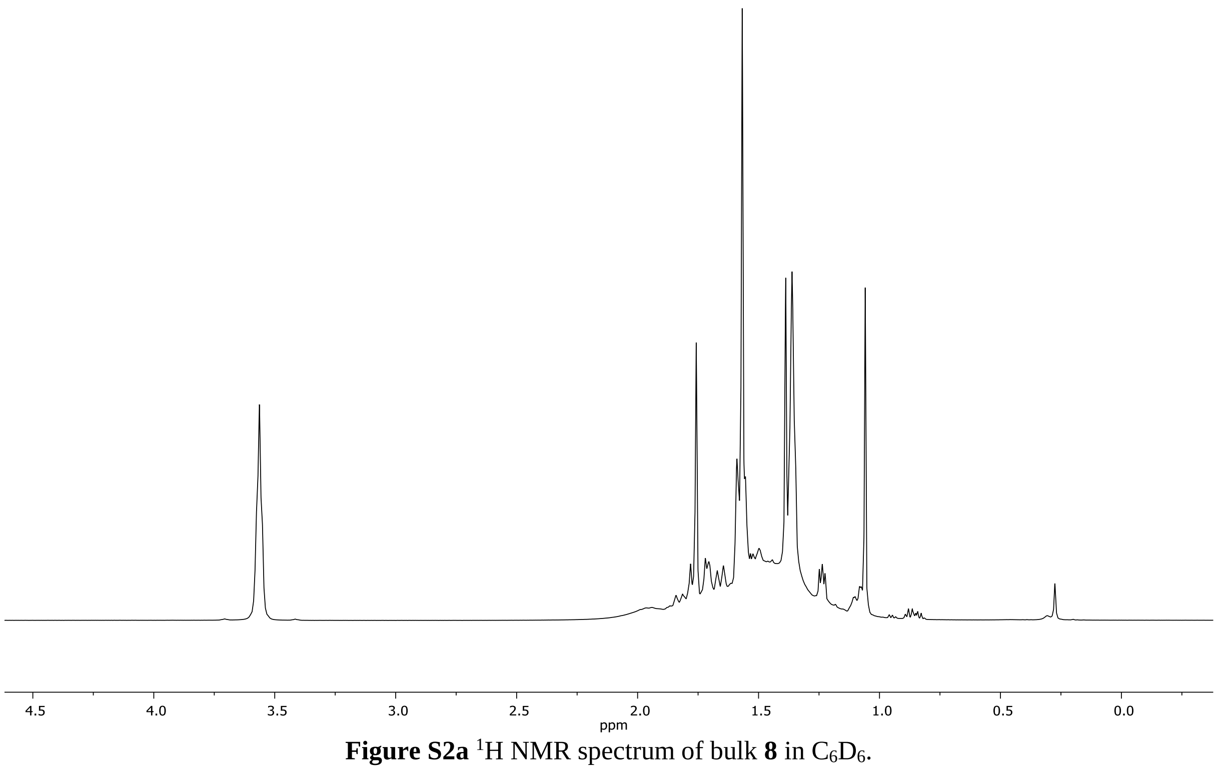

Figure S2a ^{1}H NMR spectrum of bulk **8** in C_6D_6.

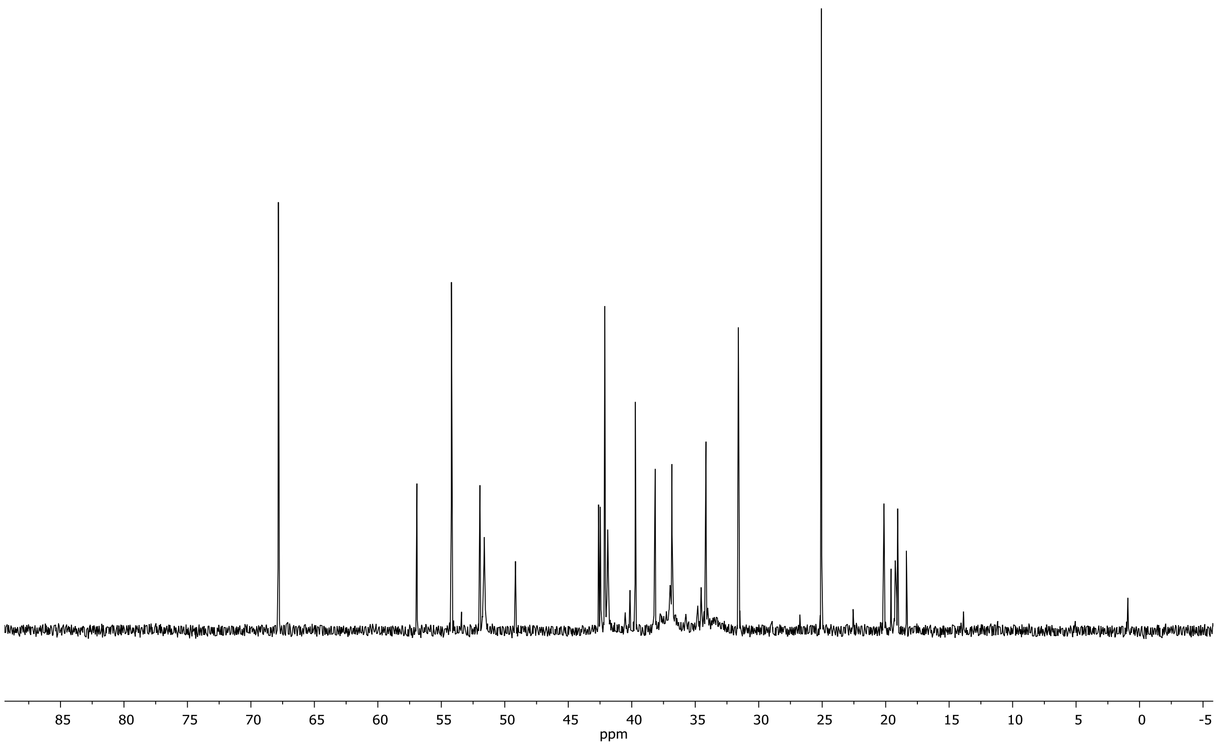

Figure S2b ^{13}C NMR spectrum of bulk **8** in C_6D_6.

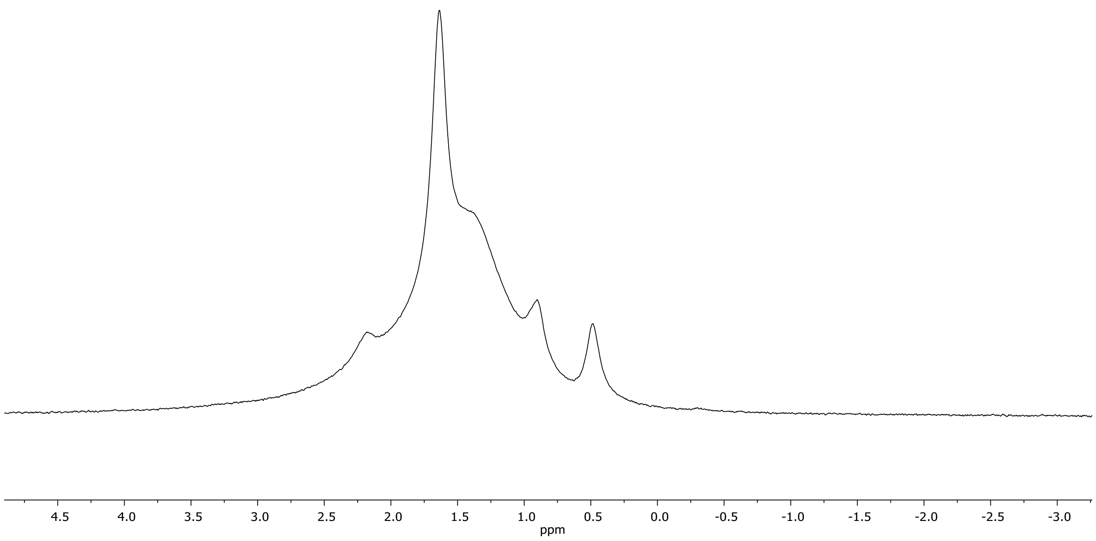

Figure S2c ^{7}Li NMR spectrum of bulk **8** in C_6D_6.

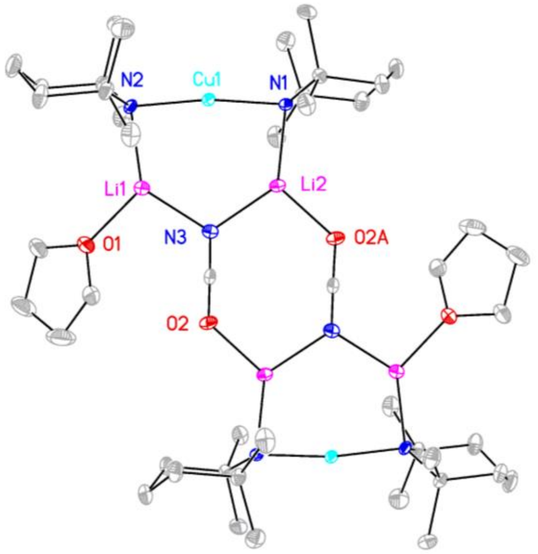

Figure S3 Molecular structure of pure **8a**$_2$ (30% probability and with H-atoms omitted). Selected bond lengths (Å) and angles (°): Cu1–N1 1.9120(17), Cu1–N2 1.9173(17), N1–Li2 2.013(4), N2–Li1 2.006(4), N3–Li1 2.020(5), N3–Li2 2.037(5), O2A–Li2 1.931(4), N3–C19 1.172(3), O2–C19 1.210(3), N1–Cu1–N2 171.47(7), Cu1–N1–Li2 86.61(14), Cu1–N2–Li1 87.43(14), Li1–N3–Li2 110.28(18).

Additional characterization of pure (TMP)$_2$Cu(OCN)Li$_2$(THF) 8a

8a crystallized as a two component non-merohedral twin. Orientation matrices for the two components were found using the program Cell Now and the two components were integrated with SAINT.[2] The exact twin law determined by the integration program was (–0.99994, 0.00004, 0.00042), (–0.00003, –1.00011, –0.00063), (0.32832, 0.36100, 1.00005). The data were corrected for absorption using Twinabs.[2] 6927 reflections (2275 unique) involved domain 1 only (mean I/σ = 46.9), 6872 reflections (2247 unique) involved domain 2 only (mean I/σ = 15.6) and 5721 reflections (2638 unique) involved two domains (mean I/σ = 42.9). The structure was solved with SHELXT[3] using data from domain 1 only (HKLF 4) and for refinement, overlaps were also included (HKLF 5).

2. *APEX3*, Bruker AXS Inc., Madison, Wisconsin, USA., 2016.
3. G. M. Sheldrick, *Acta Crystallogr., Sect. A: Found. Crystallogr.*, 2015, **71**, 3-8.

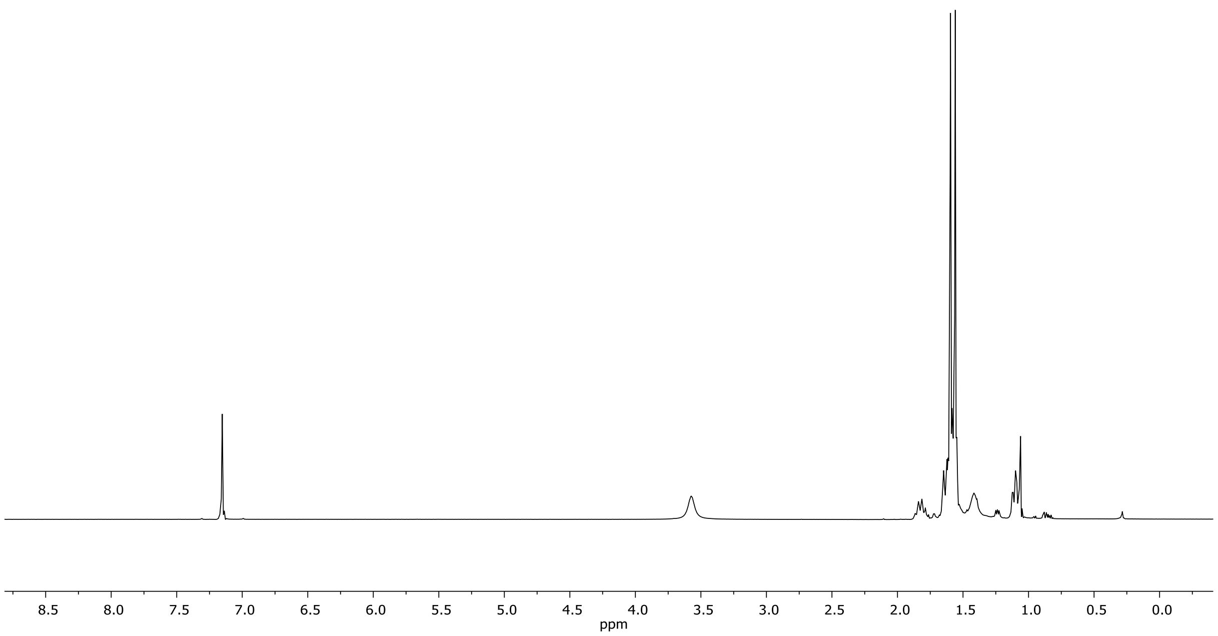

Figure S4a ^{1}H NMR spectrum of **8a** in C_6D_6.

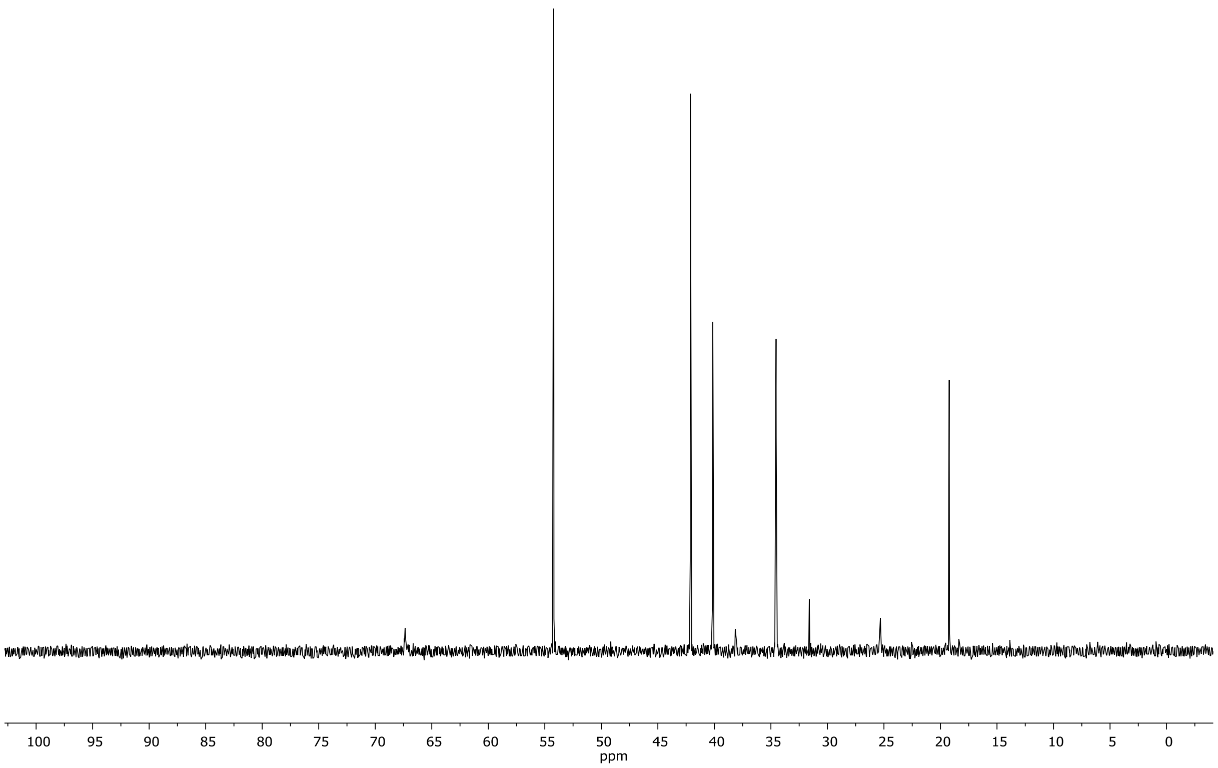

Figure S4b ^{13}C NMR spectrum of **8a** in C_6D_6.

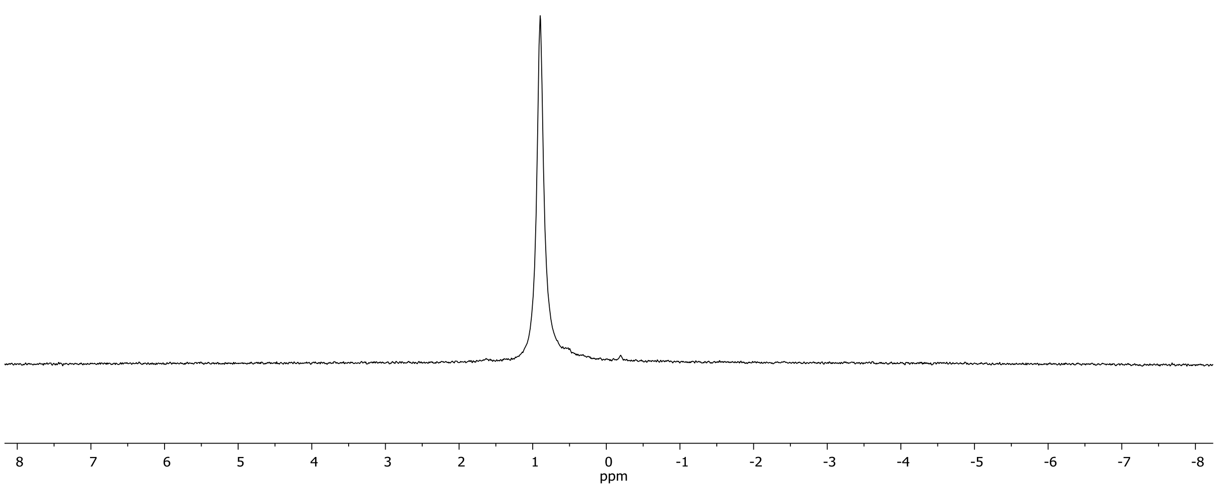

Figure S4c ^{7}Li NMR spectrum of **8a** in C_6D_6.

Additional characterization of 9

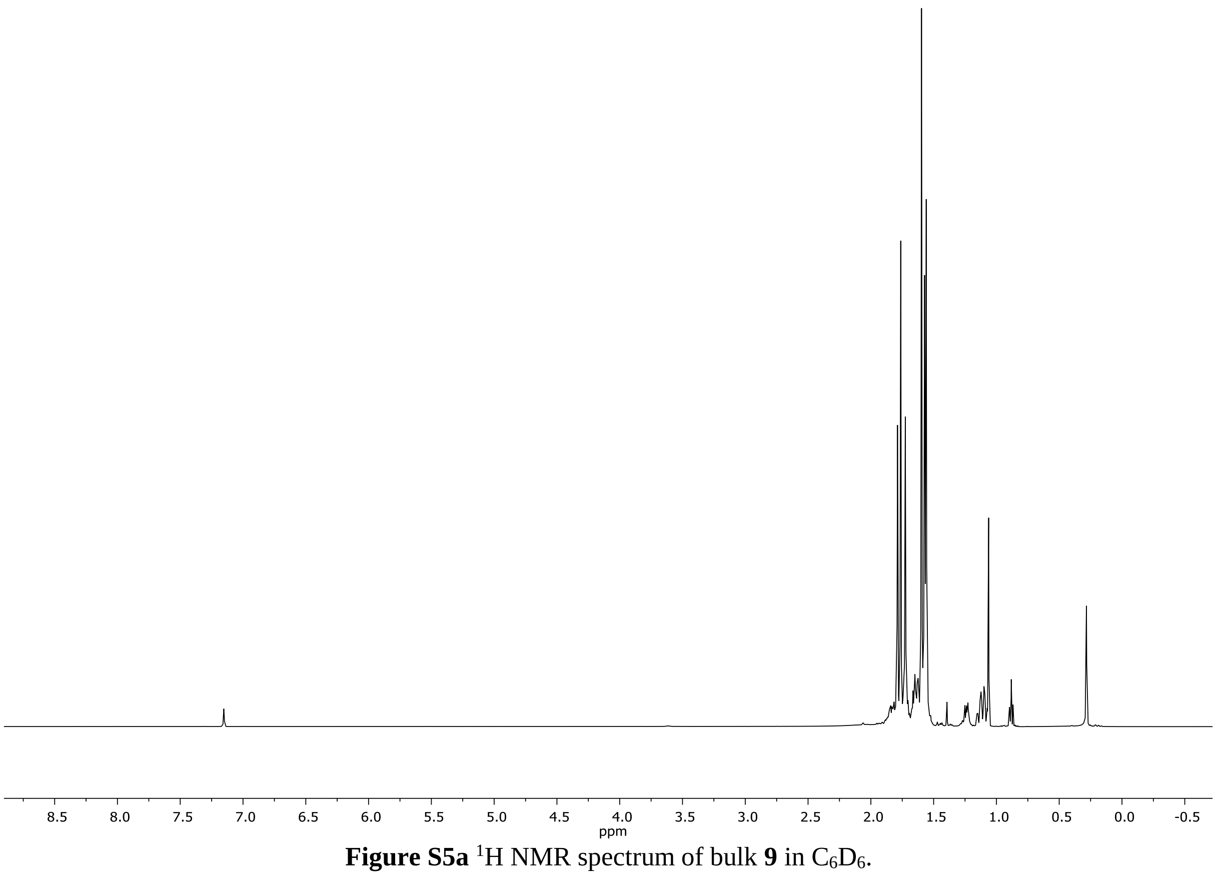

Figure S5a ^{1}H NMR spectrum of bulk **9** in C_6D_6.

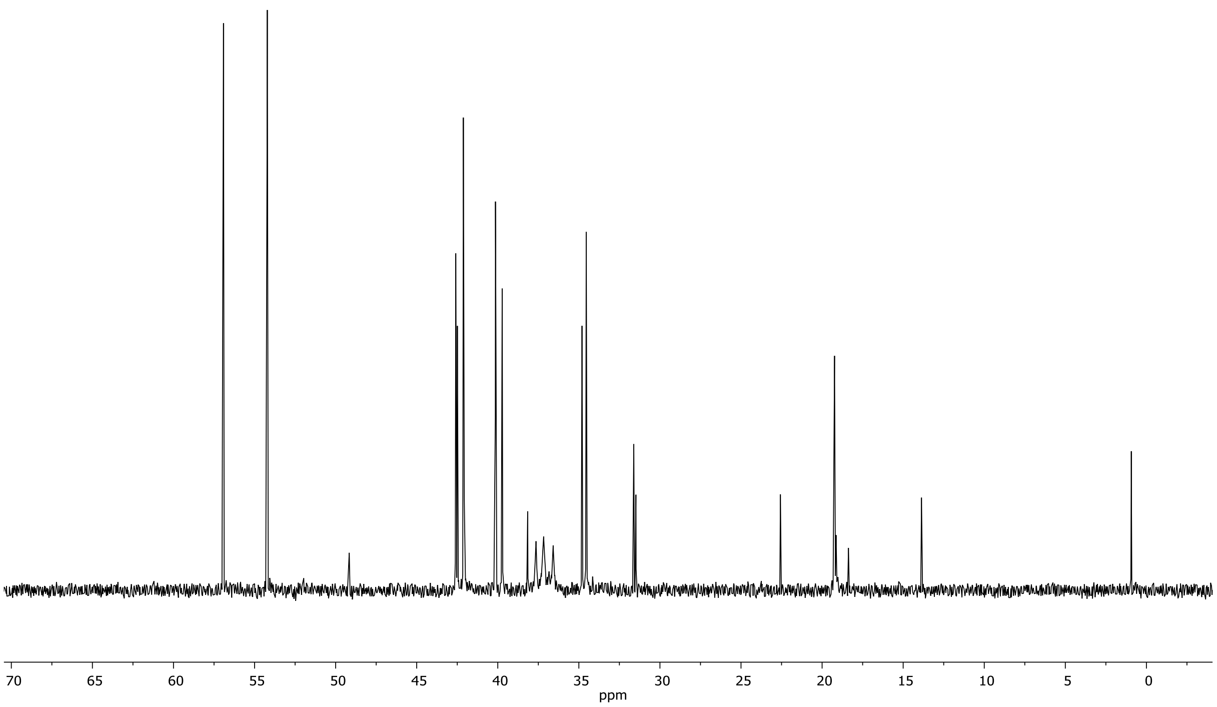

Figure S5b ^{13}C NMR spectrum of bulk **9** in C_6D_6.

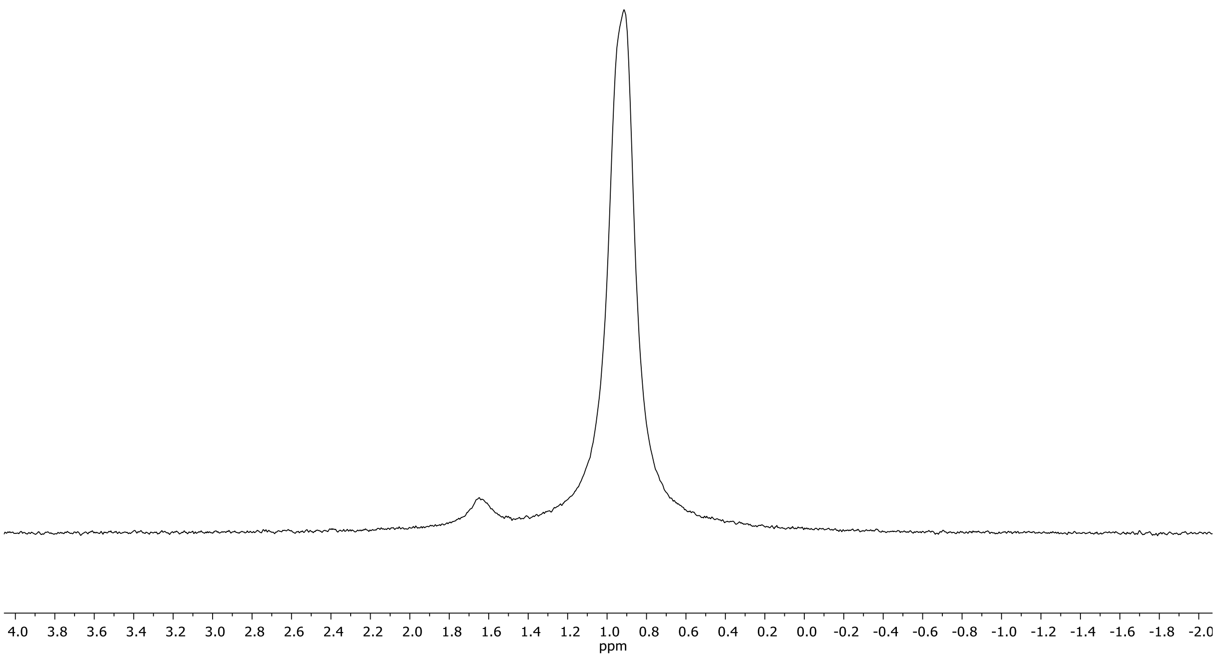

Figure S5c ^{7}Li NMR spectrum of bulk **9** in C_6D_6.

Additional characterization of TMP$_2$CuLi 9a

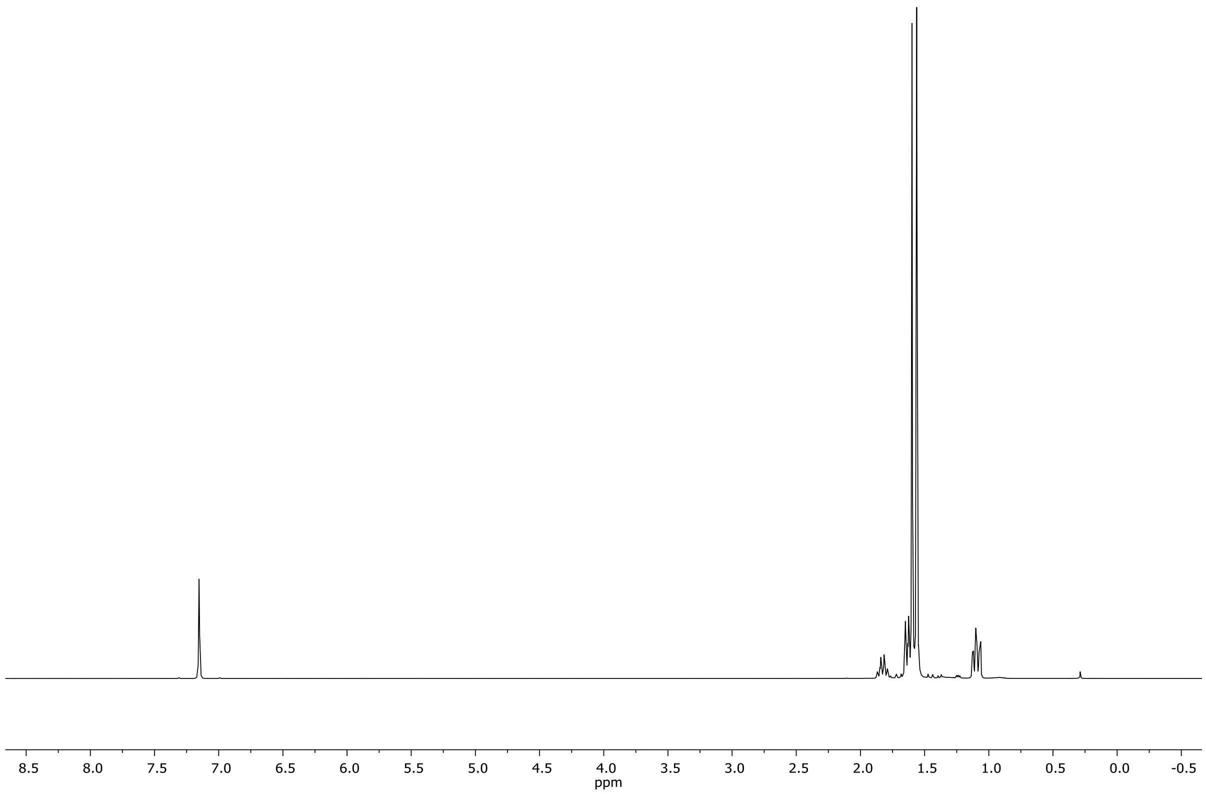

Figure S6a ^{1}H NMR spectrum of **9a** in C_6D_6.

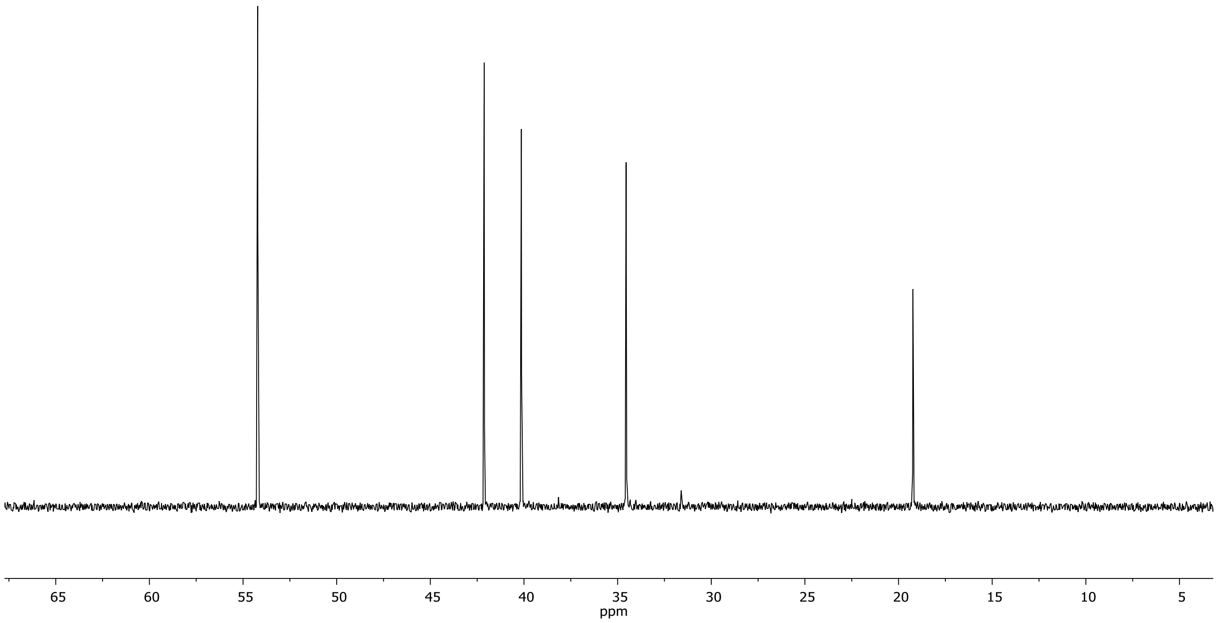

Figure S6b ^{13}C NMR spectrum of **9a** in C_6D_6.

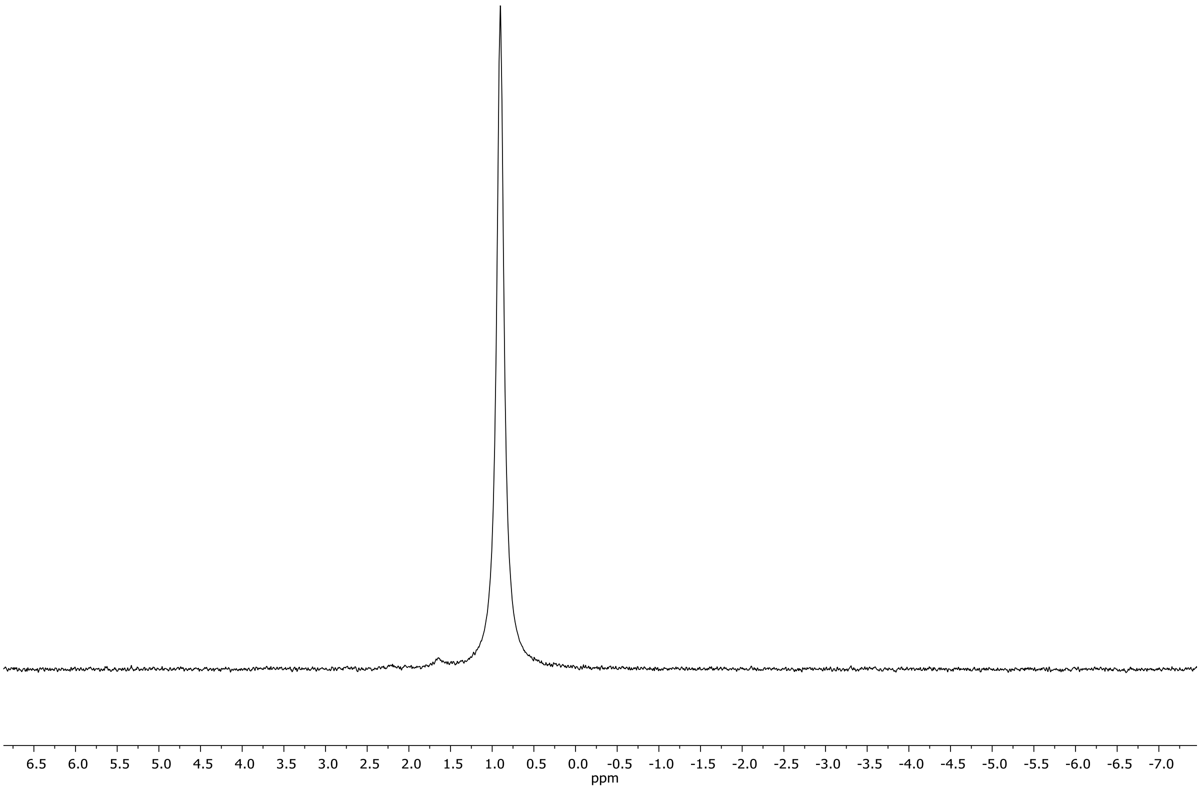

Figure S6c ^{7}Li NMR spectrum of **9a** in C_6D_6.

Synthesis and characterization of TMPLi 9b[4]

*n*BuLi (2.5 mL, 1.6 M in hexanes, 4 mmol) was added to a stirred solution of TMPH (0.68 mL, 4 mmol) in hexane (4 mL) at –78°C. The solution was returned to room temperature to give a pale yellow solution. Storage at –27°C for 12 hrs gave light yellow block like crystals. Yield 247 mg (42%), melting point 196°C. Elemental Analysis, $C_9H_{18}LiN$ requires (%) C 73.44, H 12.33, N 9.52; found (%) C, 72.82; H, 12.41; N, 9.85. ^{1}H NMR (500 MHz, 298 K, C_6D_6) δ 1.77 (m, 2H, TMP-4-tet), 1.72 (m, 0.66H, TMP-4-tri), 1.53 (m, 0.25H, TMPH-4), 1.37 (m, 4H, TMP-3,5-tet), 1.36 (br, 1.35 (s, 12H, TMP-Me-tet), 1.30 (s, 4H, TMP-Me-tri), 1.29 (m, 1.33H, TMP-3,5-tri), 1.24 (m, 0.5H, TMPH-3,5), 1.06 (s, 1.5H, TMPH-Me). ^{13}C NMR (125 MHz, 298 K, C_6D_6) δ 52.0 (TMP-2,6-tet), 51.9 (TMP-2,6-tri), 49.2 (TMPH-2,6), 42.7 (TMP-3,5-tri), 42.4 (TMP-3,5-tet), 38.2 (TMPH-3,5), 36.6 (TMP-Me-tri), 36.5 (TMP-

Me-tet), 31.6 (TMPH-Me), 19.7 (TMP-4-tet), 19.4 (TMP-4-tri), 18.4 (TMPH-4). ^{7}Li NMR (194 MHz, 298 K, C_6D_6) δ 2.24 (s).

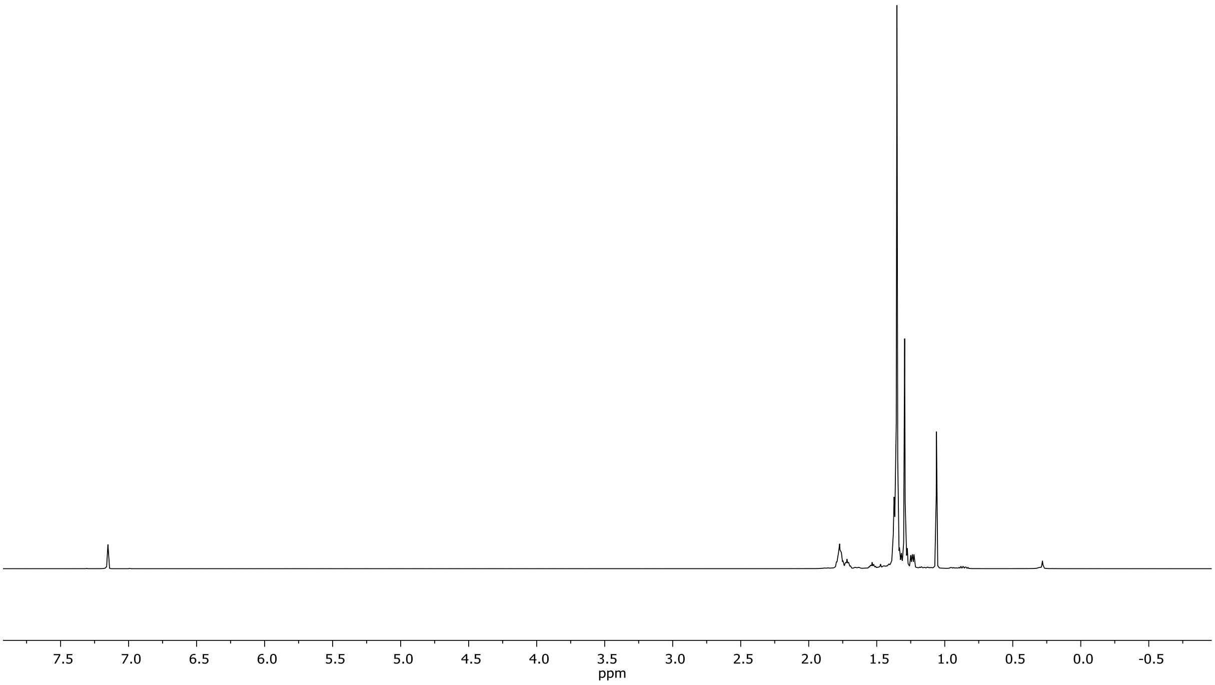

Figure S7a ^{1}H NMR spectrum of **9b** in C_6D_6.

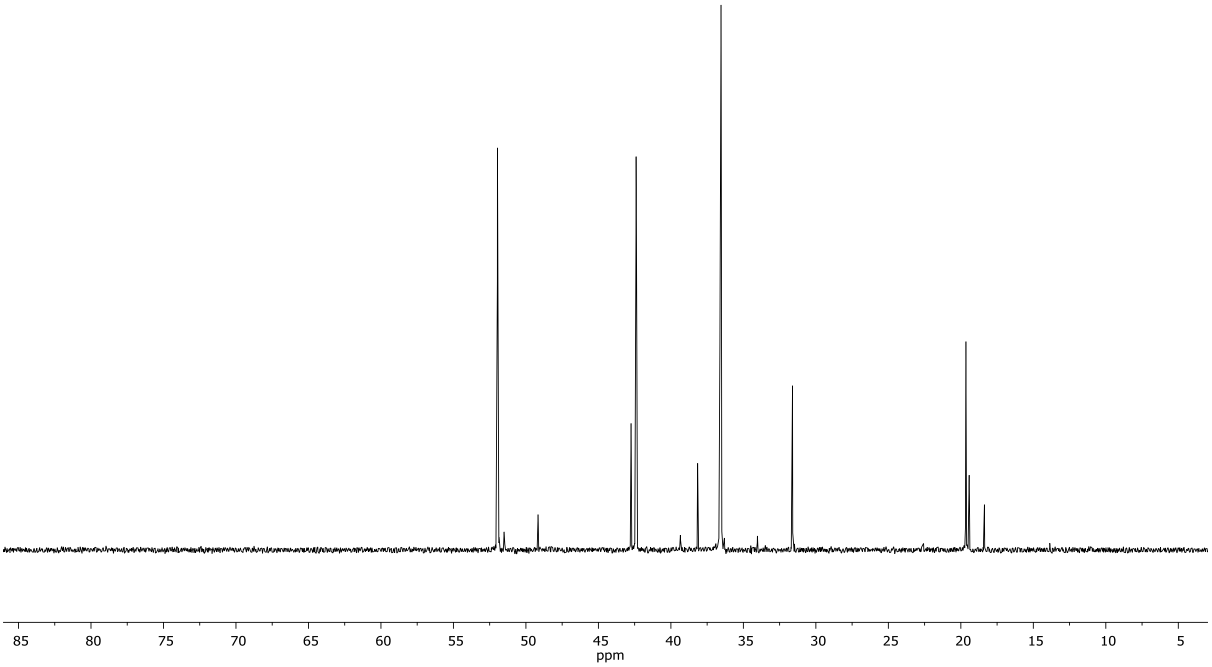

Figure S7b ^{13}C NMR spectrum of **9b** in C_6D_6.

4. E. Hevia, A. R. Kennedy, R. E. Mulvey, D. L. Ramsay, S. D. Robertson, *Chem. Eur. J.*, 2013, **19**, 14069-14075.

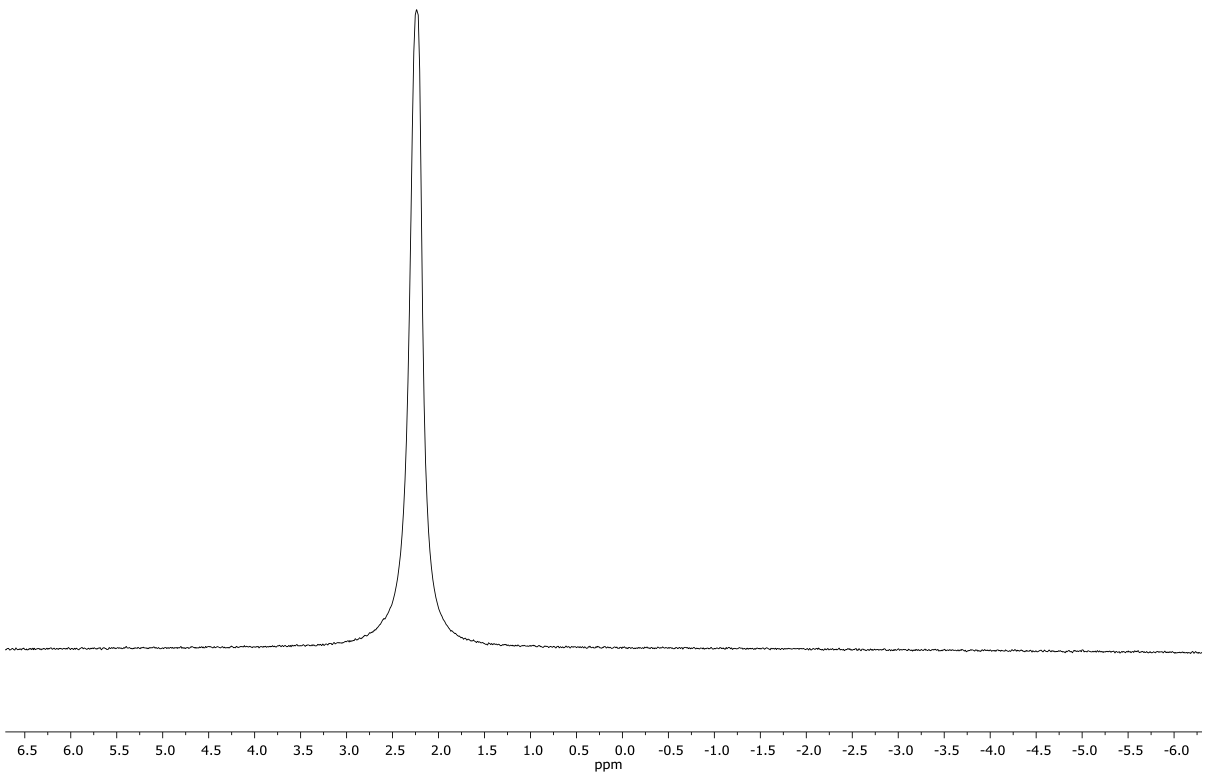

Figure S7c ^{7}Li NMR spectrum of **9b** in C_6D_6.

Synthesis and characterization of TMPCu 9c

*n*BuLi (2.5 mL, 1.6 M in hexanes, 4 mmol) was added to a solution of TMPH (0.68 mL, 4 mmol) in hexane/THF (1:1, 6 mL) at -78 °C. The solution was warmed to room temperature and transferred to a suspension of CuCl (0.40 g, 4 mmol) in hexane/THF (1:1, 6 mL), at -78 °C. The dark suspension was warmed to room temperature and heated to reflux, whereupon it was filtered to give a yellow suspension. The solid was dissolved with gentle warming and allowed to crystallize at room temperature. Yield 147 mg (18%), melting point 236°C. Elemental Analysis, $C_9H_{18}CuN$ requires (%) C 53.04, H 8.90, N 6.87; found (%) C, 52.78; H, 8.78; N, 6.76. ^{1}H NMR (500 MHz, C_6D_6): δ 1.70 (s, 12H, TMP-Me), 1.60 (m, 2H, TMP-4), 1.40 (m, 4H, TMP-3,5). ^{13}C NMR (125 MHz, C_6D_6): δ 56.5 (TMP-2,6), 41.8 (TMP-3,5), 37.9 (TMP-Me), 18.6 (TMP-4).

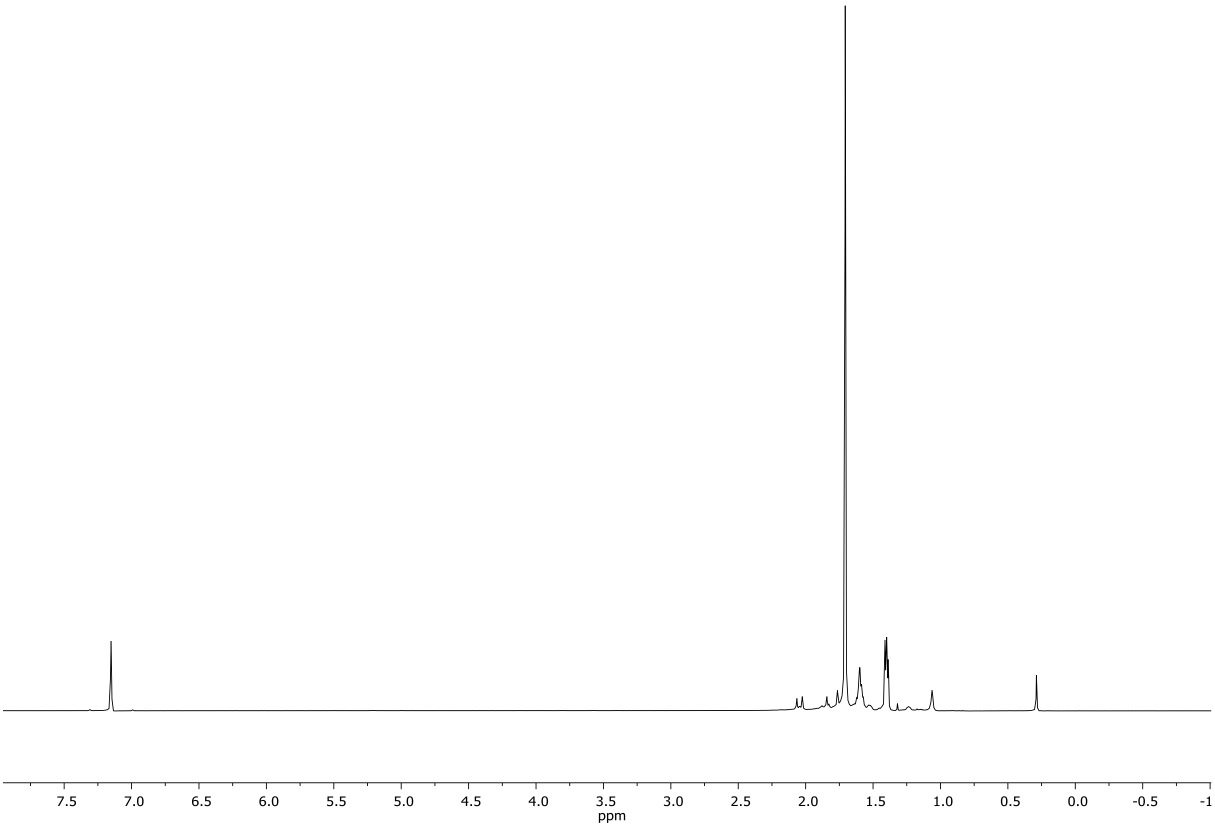

Figure S8a ^{1}H NMR spectrum of **9c** in C_6D_6.

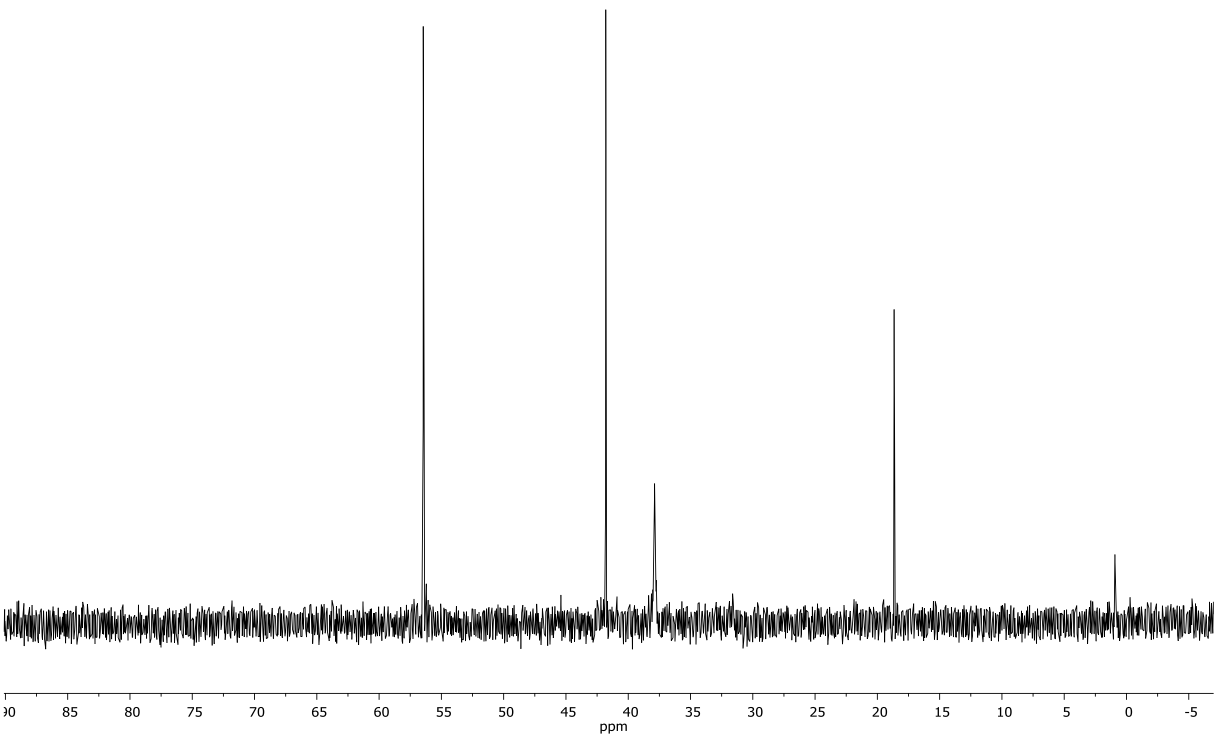

Figure S8b ^{13}C NMR spectrum of **9c** in C_6D_6.

Synthesis and characterization of ($TMPH_2$)OCN 10

Conc. sulfuric acid (0.26 mL, 5 mmol) was added dropwise to a solution of TMPH (1.68 mL, 10 mmol) in ethanol (absolute, 50 mL). The solution was concentrated *in vacuo* and Et_2O added until precipitation occurred. These white microcrystals of $(TMPH_2)_2SO_4$ were collected by filtration and washed with Et_2O (3 x 5 mL). A solution of $(TMPH_2)_2SO_4$ (0.76 g, 2 mmol) in water (10 mL) was added to **6** (0.44 g, 2 mmol) in water (10 mL), whereupon a white precipitate formed immediately. The suspension was stirred for 10 minutes and filtered twice. The solvent was removed *in vacuo* and the product extracted in ethanol (absolute, 20 mL). The solution was concentrated and Et_2O slowly added until crystallization initiated. The crystalline product was collected by filtration and washed with Et_2O (3 x 5 mL) to give ($TMPH_2$)OCN **10**. Yield 0.34 g (46 %), melting point >300 °C. Elemental Analysis, $C_{10}H_{20}N_2O$ requires (%) C 65.18, H 10.94, N 15.20; found (%) C 64.26, H 10.99, N 14.54. ^1H NMR spectroscopy (500 MHz, CD_3OD) δ 1.80 (m, 2H, TMP-4), 1.66 (m, 4H, TMP-3,5), 1.43

(s, 12H, TMP-Me). ^{13}C NMR spectroscopy (125 MHz, CD_3OD) δ 130.3 (OCN), 57.8 (TMP-2,6), 36.2 (TMP-3,5), 27.8 (TMP-Me), 17.3 (TMP-4). Selected IR spectroscopy (nujol) $\bar{\nu}$ 3020-2350(w, br, NH), 2134(s, CN), 1601(m, CO) cm^{-1}.

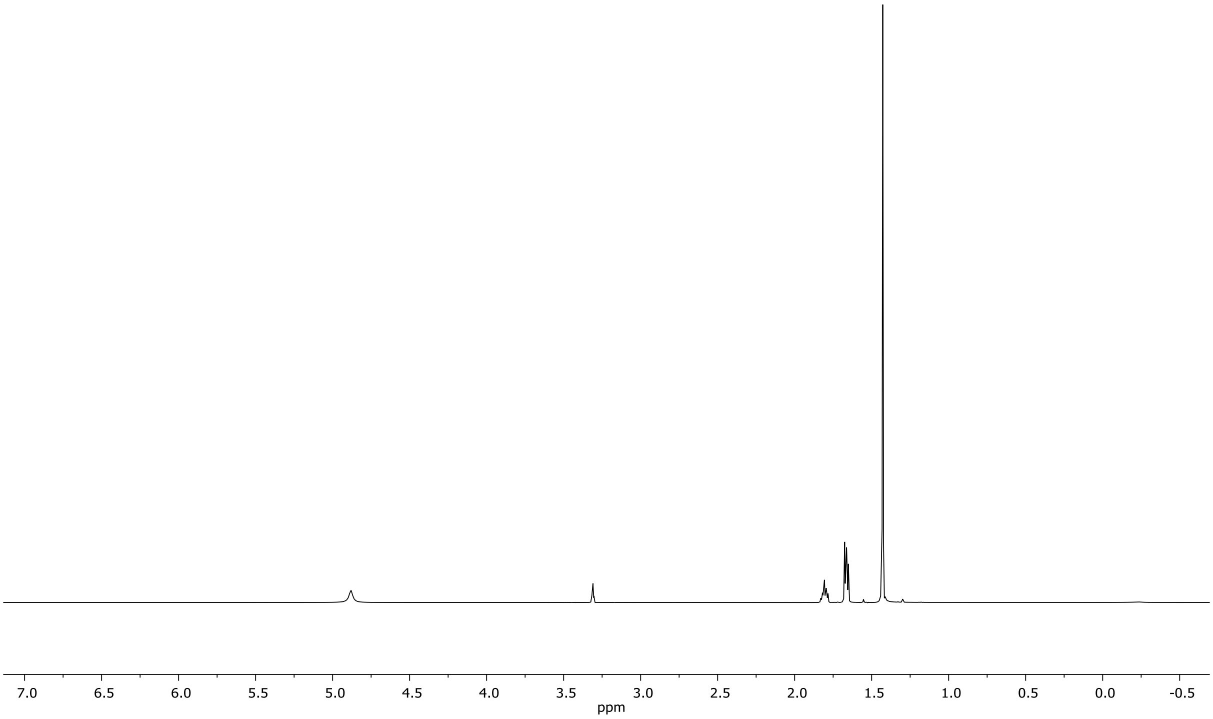

Figure S9a ^{1}H NMR spectrum of **10** in CD_3OD.

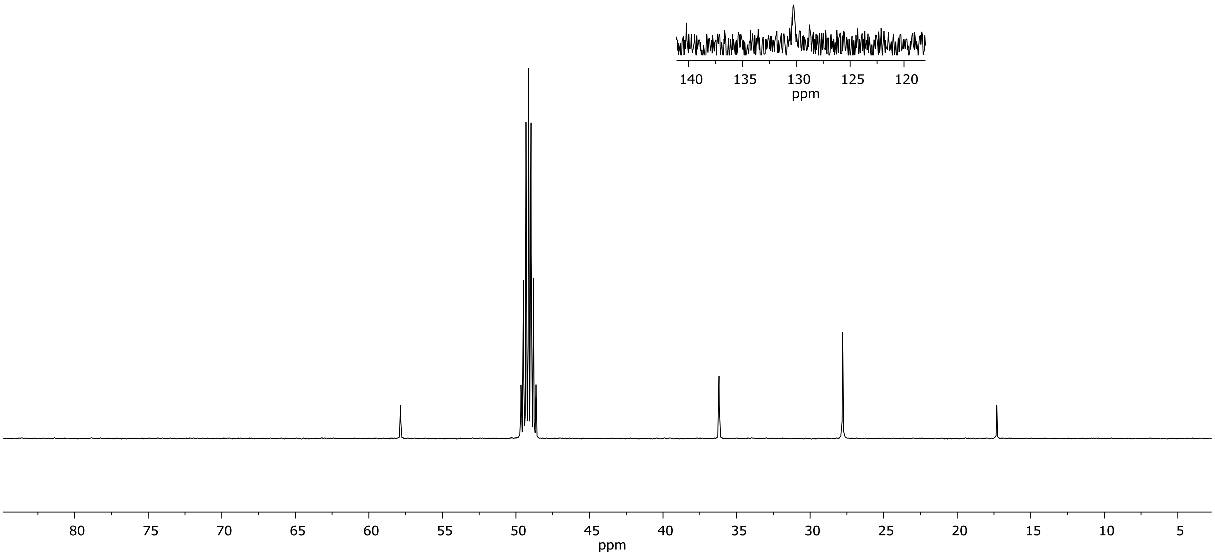

Figure S9b ^{13}C NMR spectrum of **10** in CD_3OD. Inset: OCN region of the spectrum.

Additional characterization of $(TMP)_2(OCN)Li_3(THF)_2$ 11

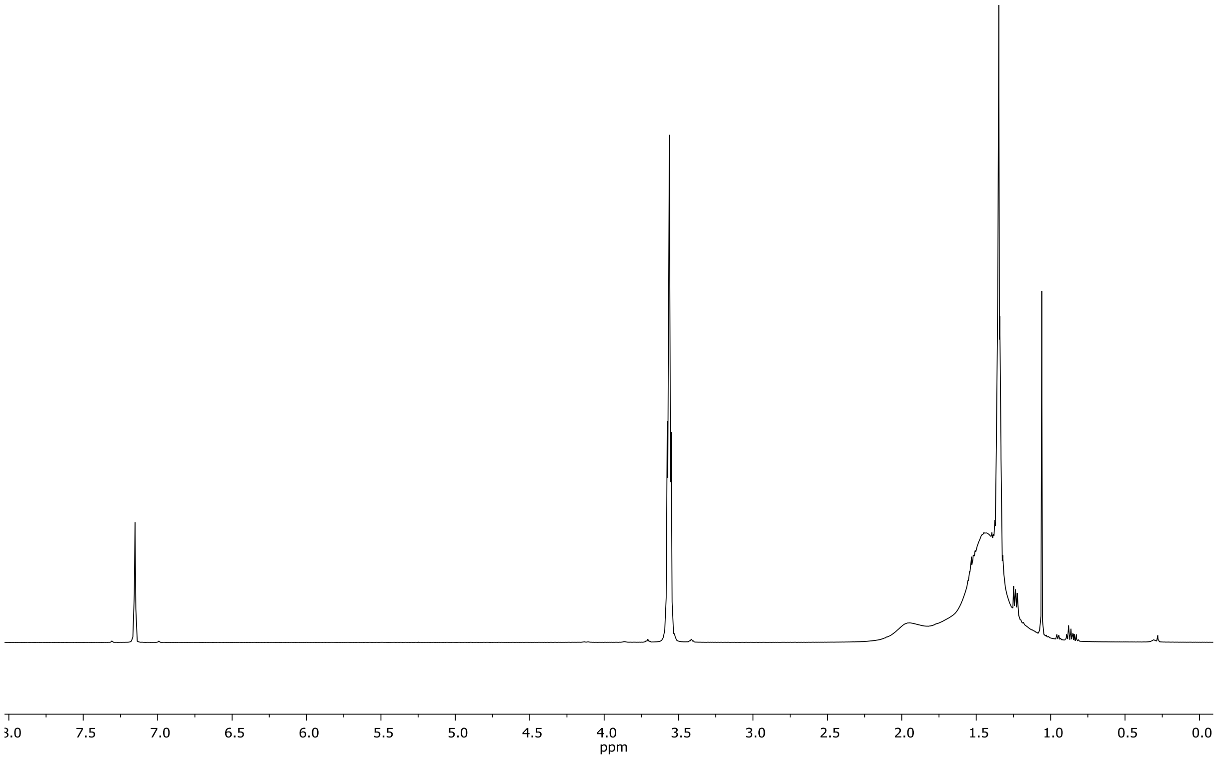

Figure S10a ^{1}H NMR spectrum of **11** in C_6D_6.

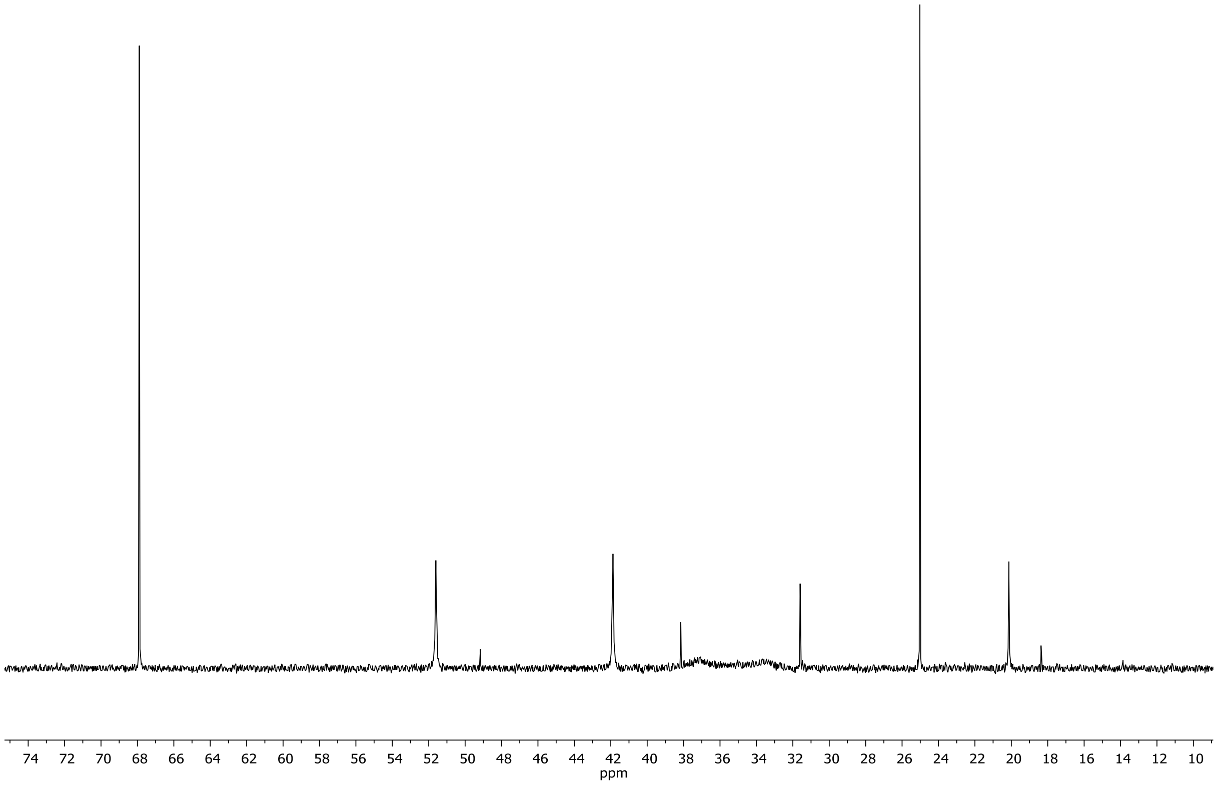

Figure S10b ^{13}C NMR spectrum of **11** in C_6D_6.

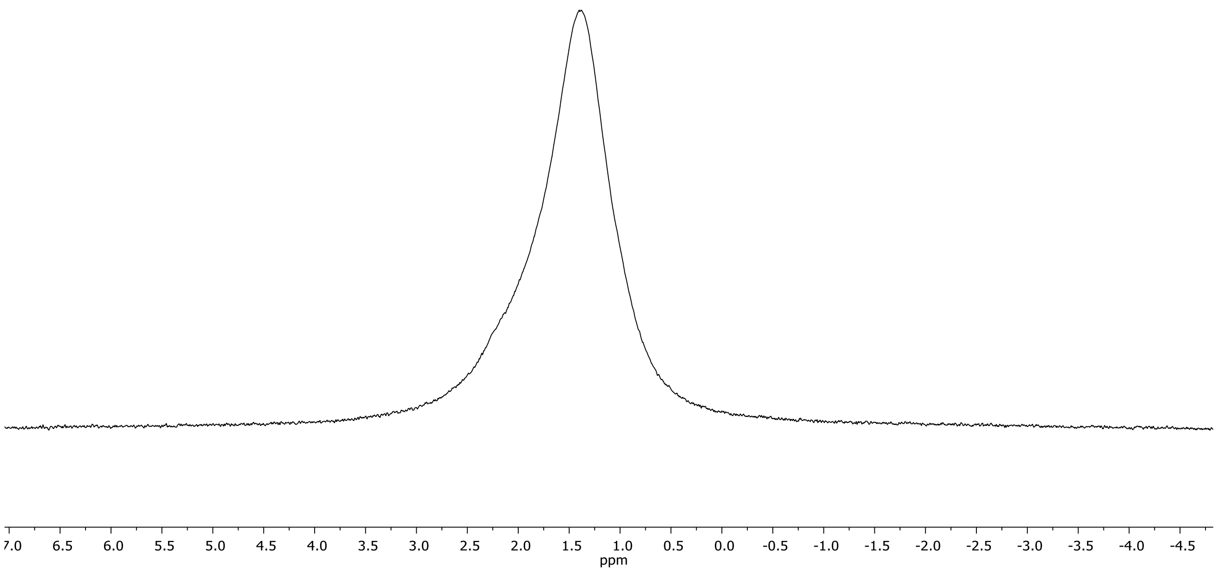

Figure S10c ^{7}Li NMR spectrum of **11** in C_6D_6.

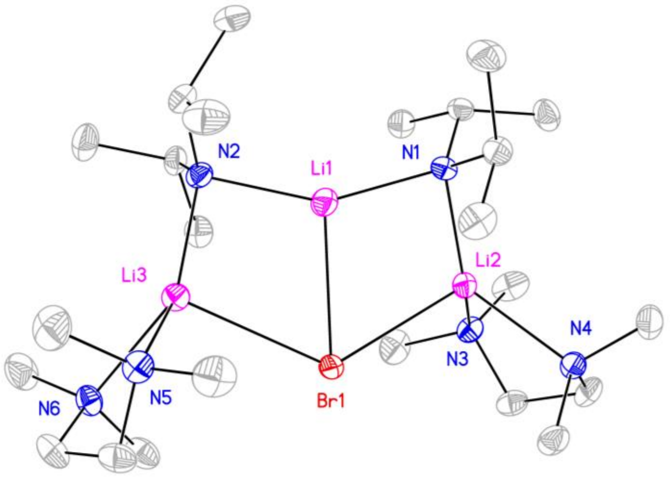

Figure S4 Molecular structure of pure **12b** (30% probability and with H-atoms omitted). Selected bond lengths (Å) and angles (°):N1–Li1 1.977(5), N2–Li1 2.024(5), Br1–Li1 2.601(5), N1–Li2 2.038(5), N2–Li3 2.019(5), Br1–Li2 2.650(4), Br1–Li3 2.708(5), N1–Li2–Br1 100.07(19), N2–Li3–Br1 101.05(18), Li2–Br1–Li3 126.46(14).

Additional characterization of (DA)$_2$CuBrLi$_2$(TMEDA)$_2$ 12b

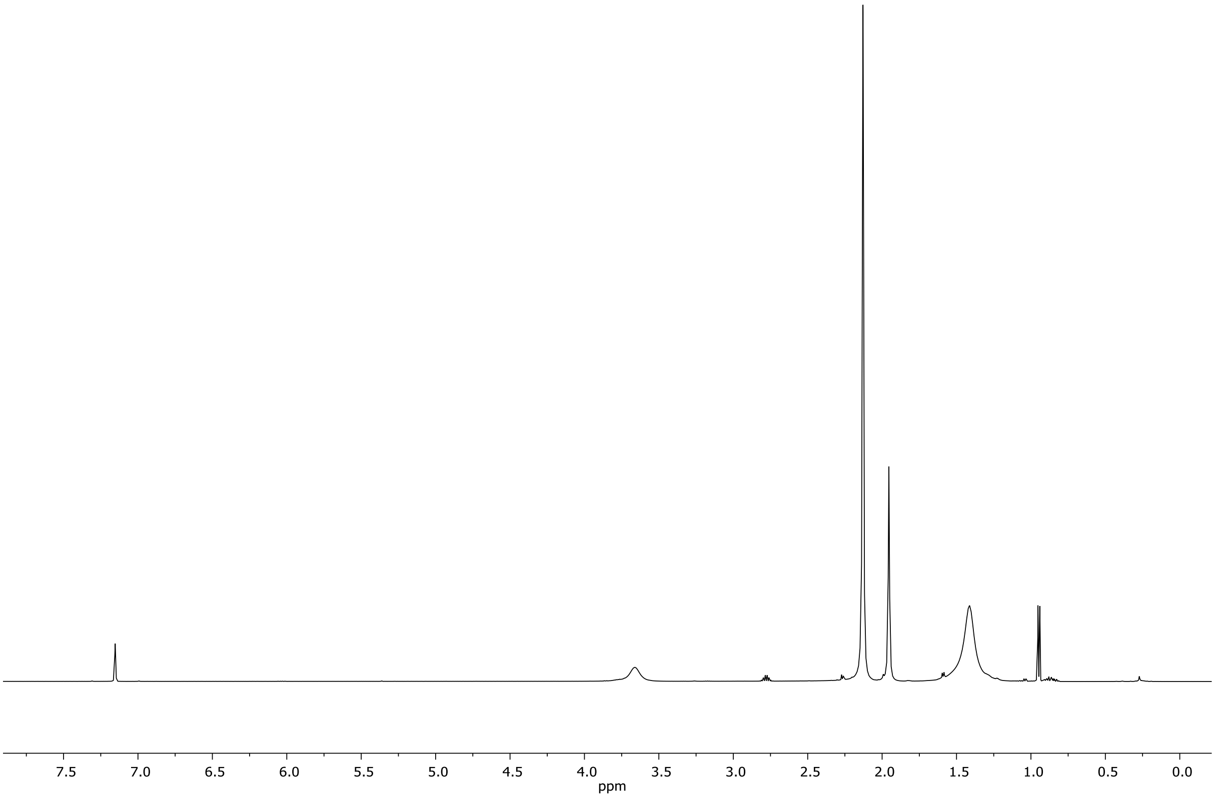

Figure S11a ^{1}H NMR spectrum of **12b** in C_6D_6.

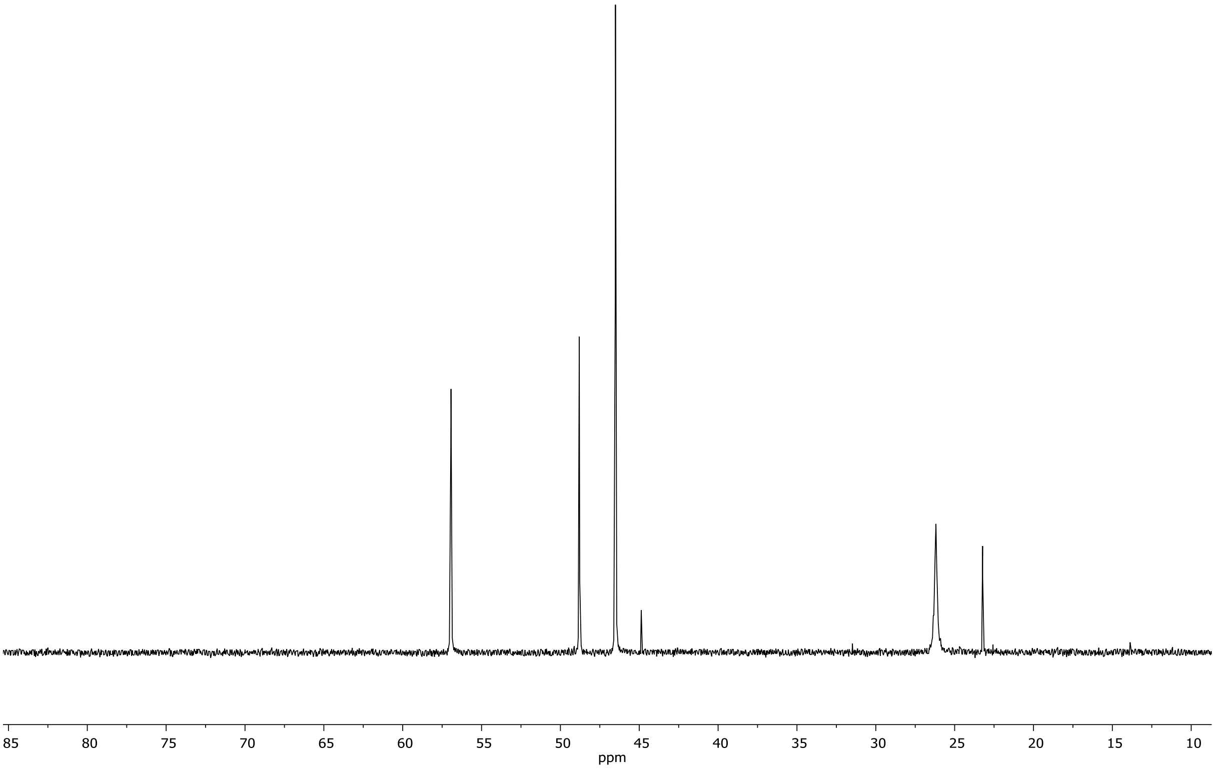

Figure S11b ^{13}C NMR spectrum of **12b** in C_6D_6.

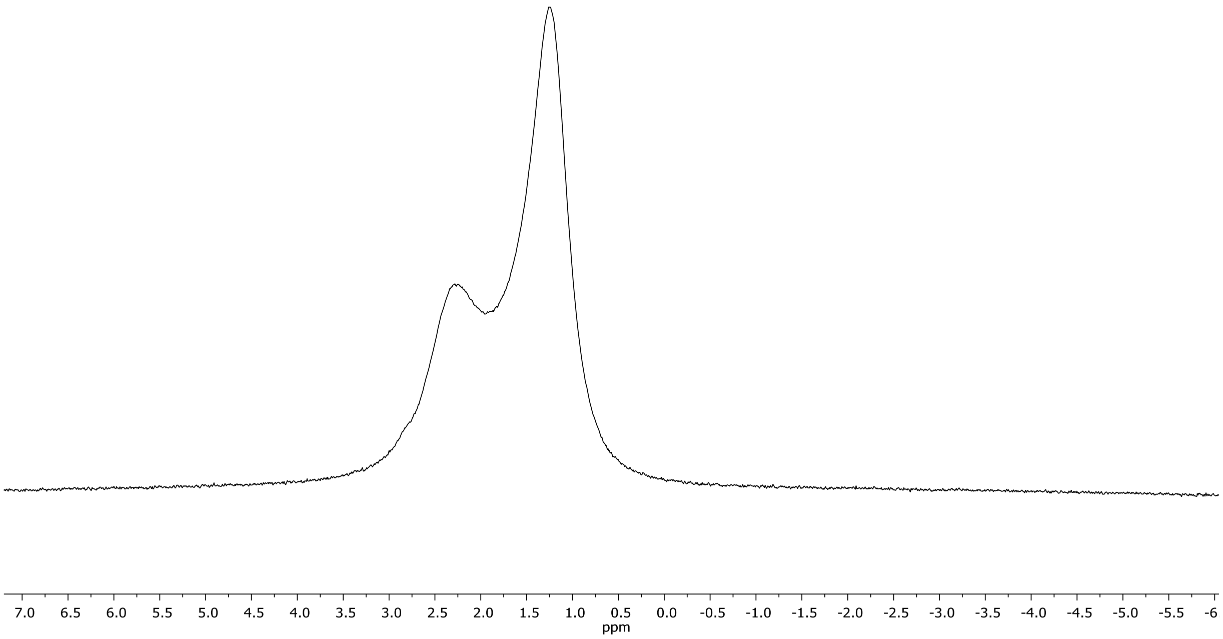

Figure S11c ^{7}Li NMR spectrum of **12b** in C_6D_6.

Additional characterization of $(DA)_2Cu_{0.09}Li_{0.91}BrLi_2(TMEDA)_2$ 12

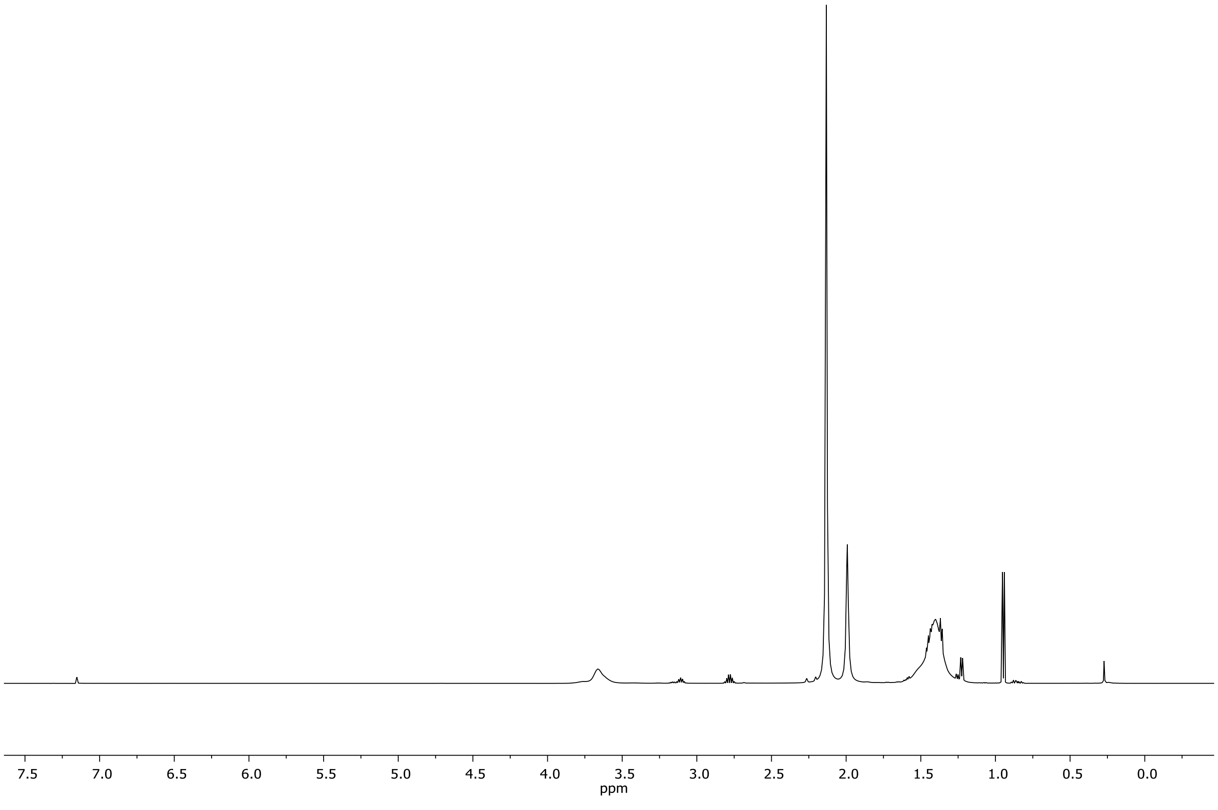

Figure S12a ^{1}H NMR spectrum of **12** (representative sample 1) in C_6D_6.

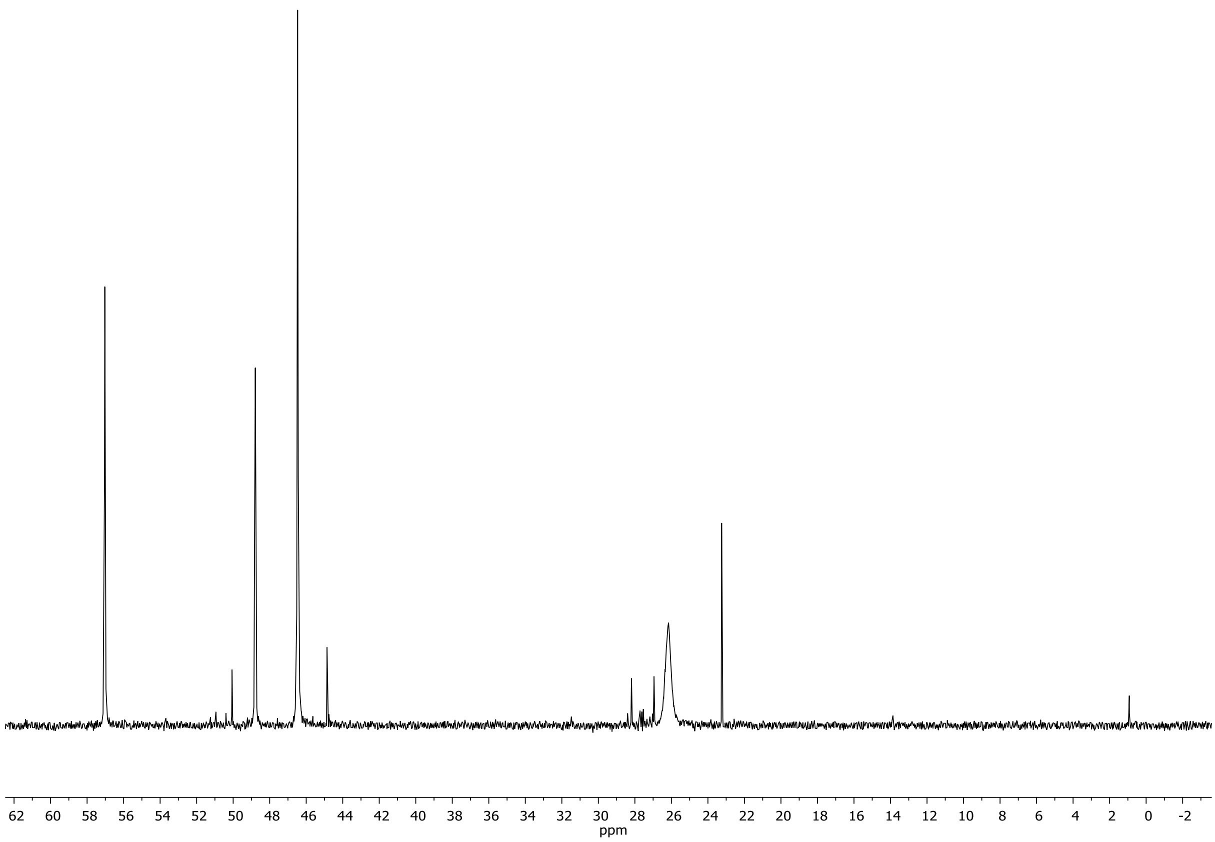

Figure S12b ^{13}C NMR spectrum of **12** (representative sample 1) in C_6D_6.

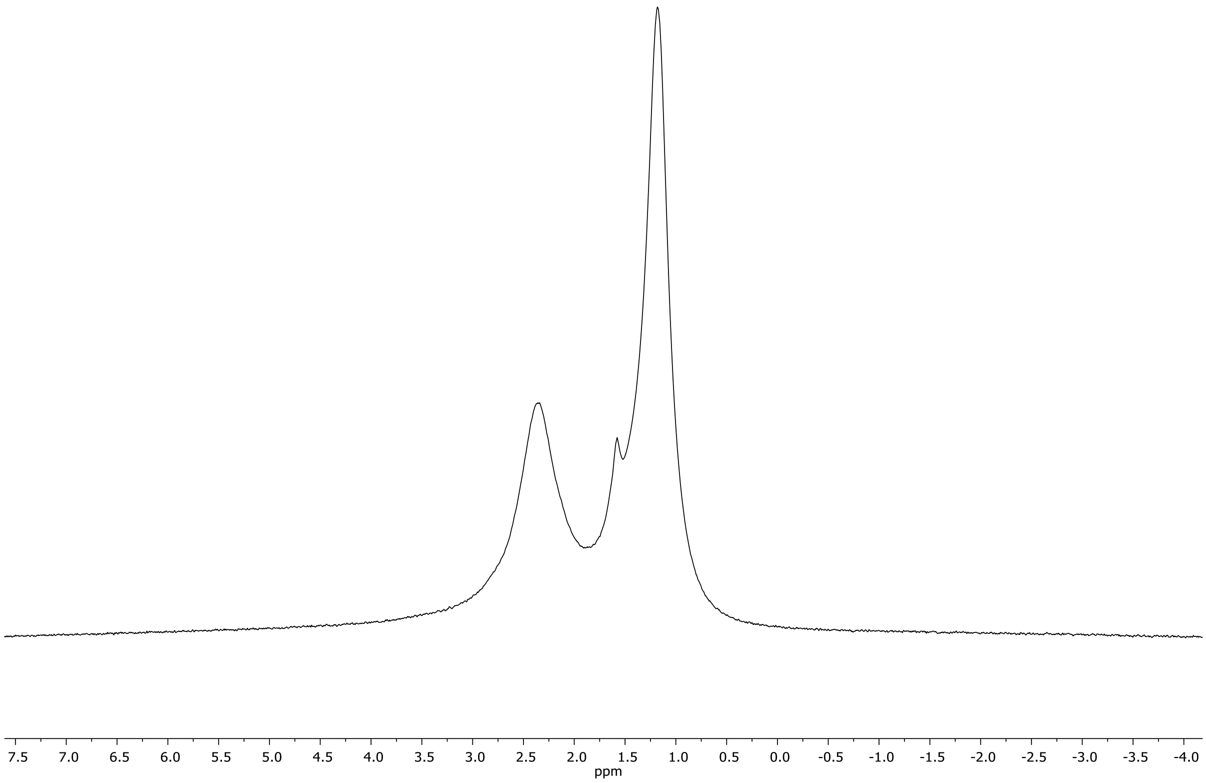

Figure S12c ^{7}Li NMR spectrum of **12** (representative sample 1) in C_6D_6.

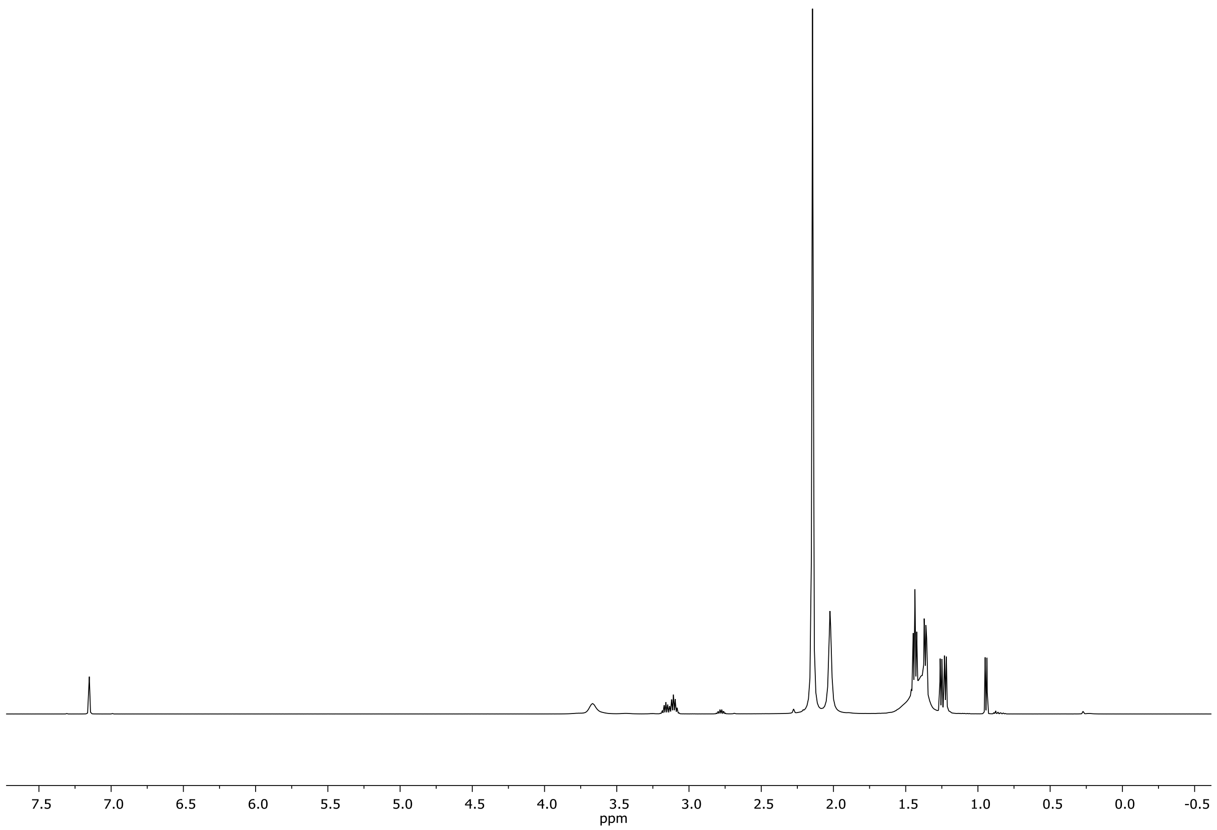

Figure S12d ^{1}H NMR spectrum of **12** (representative sample 2) in C_6D_6.

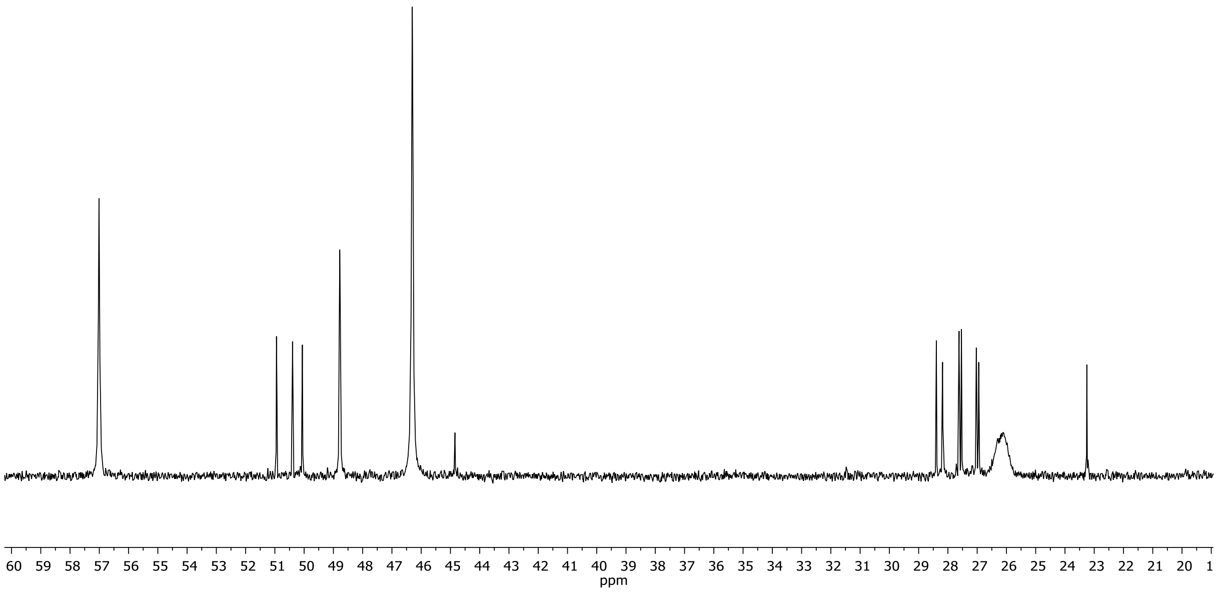

Figure S12e ^{13}C NMR spectrum of **12** (representative sample 2) in C_6D_6.

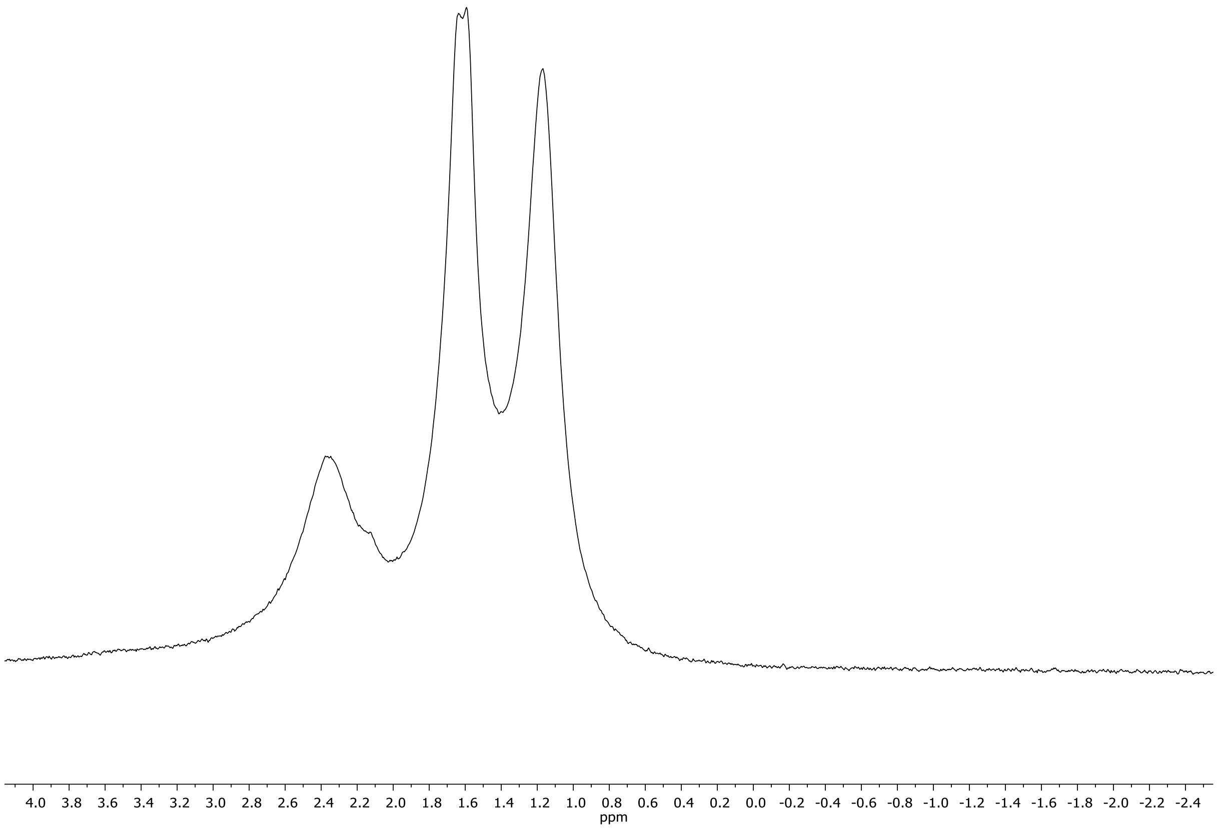

Figure S12f ^{7}Li NMR spectrum of **12** (representative sample 2) in C_6D_6.

Additional characterization of $(DA)_4Cu(OCN)Li_4(TMEDA)_2$ 13

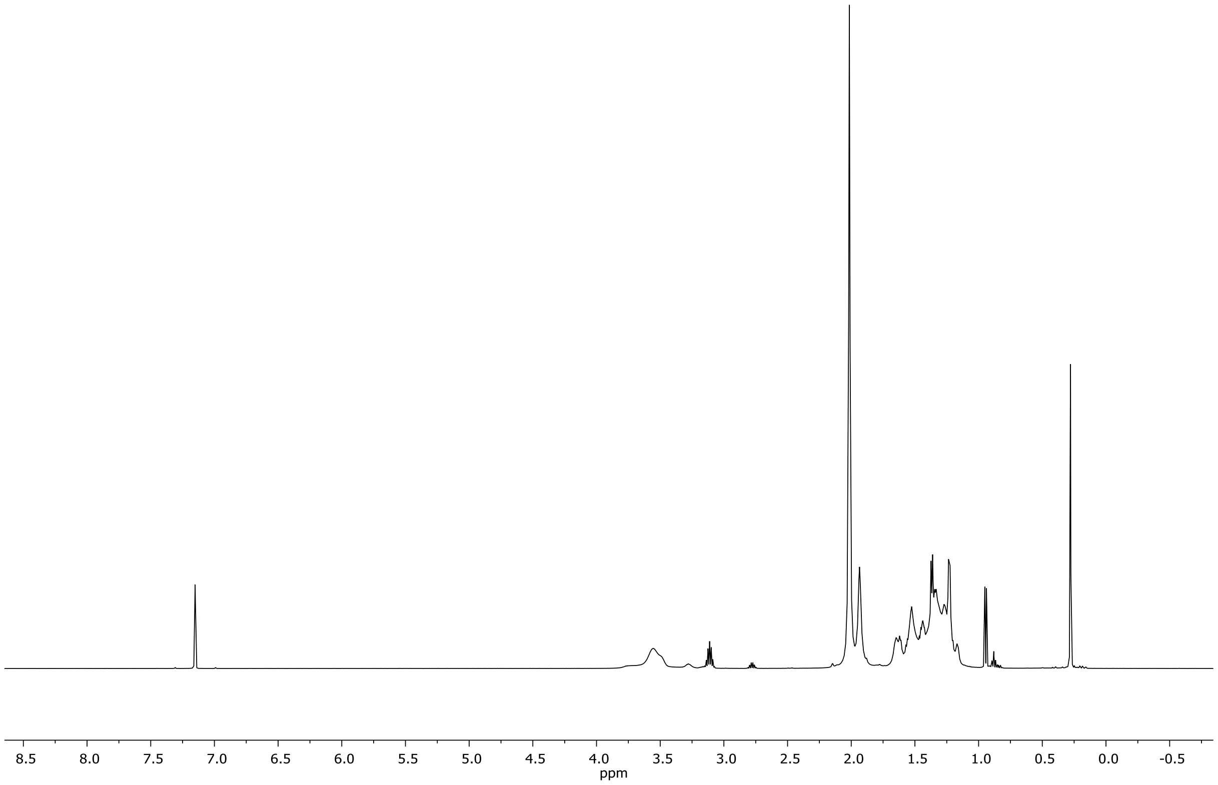

Figure S13a ^{1}H NMR spectrum of **13** in C_6D_6.

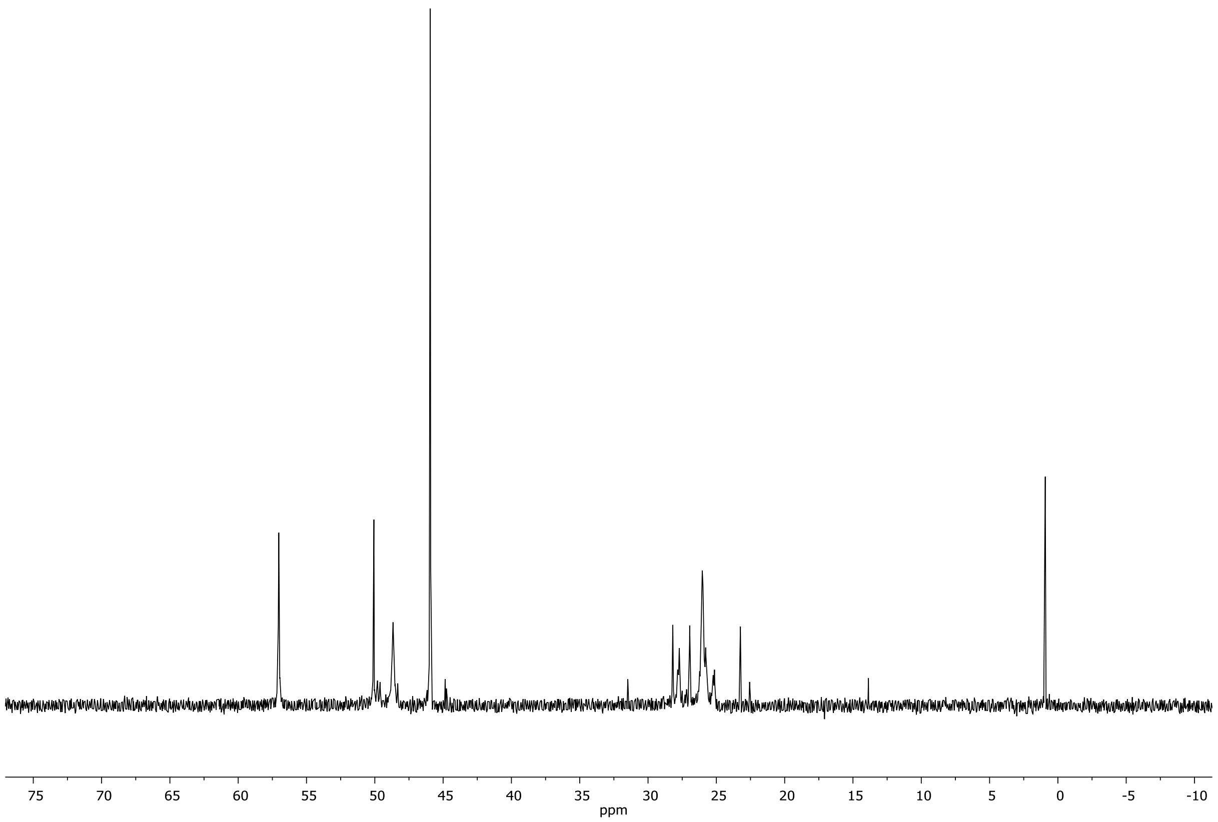

Figure S13b ^{13}C NMR spectrum of **13** in C_6D_6.

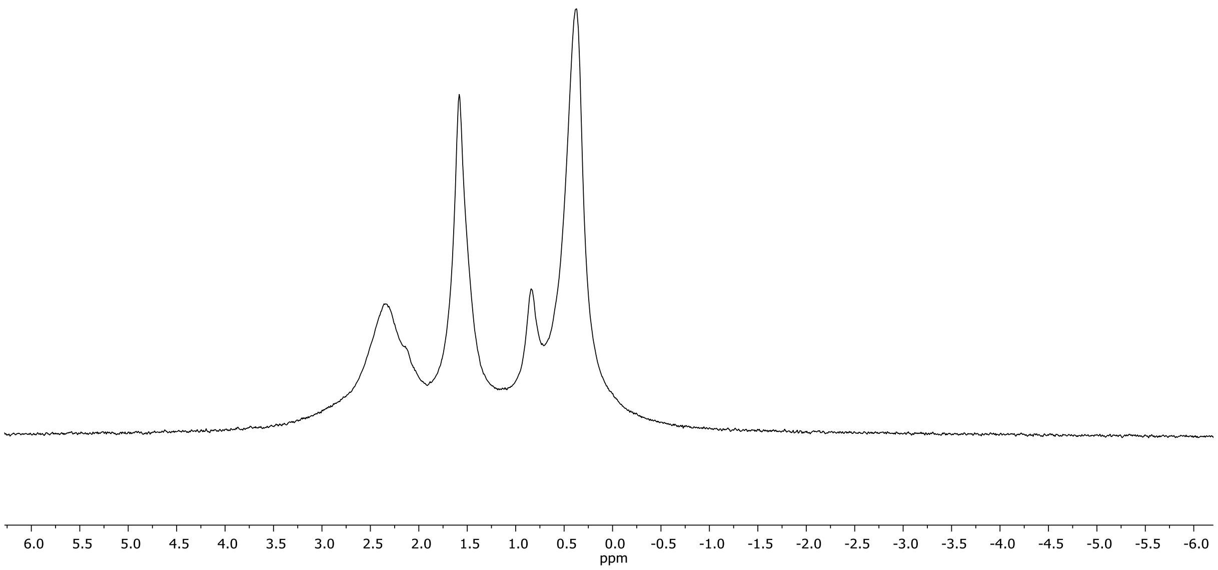

Figure S13c ^{7}Li NMR spectrum of **13** in C_6D_6.
